# Inhibitory Interplay between Orexin Neurons and Eating

**DOI:** 10.1016/j.cub.2016.07.013

**Published:** 2016-09-26

**Authors:** J. Antonio González, Lise T. Jensen, Panagiota Iordanidou, Molly Strom, Lars Fugger, Denis Burdakov

**Affiliations:** 1 Mill Hill Laboratory, The Francis Crick Institute, London NW7 1AA, UK; 2 Institute of Clinical Medicine, Aarhus University, 8200 Aarhus, Denmark; 3 Sainsbury Wellcome Centre, University College London, London W1T 4JG, UK; 4 Oxford Centre for Neuroinflammation, Division of Clinical Neurology and MRC Human Immunology Unit, Nuffield Department of Clinical Neurosciences, Weatherall Institute of Molecular Medicine, University of Oxford, Oxford OX3 9DS, UK; 5 Institute of Psychiatry, Psychology and Neuroscience, Department of Developmental Neurobiology, King’s College London, London WC2R 2LS, UK

## Abstract

In humans and rodents, loss of brain orexin/hypocretin (OH) neurons causes pathological sleepiness [1, 2, 3, 4], whereas OH hyperactivity is associated with stress and anxiety [5, 6, 7, 8, 9, 10]. OH cell control is thus of considerable interest. OH cells are activated by fasting [11, 12] and proposed to stimulate eating [13]. However, OH cells are also activated by diverse feeding-unrelated stressors [14, 15, 16, 17] and stimulate locomotion and “fight-or-flight” responses [18, 19, 20]. Such OH-mediated behaviors presumably preclude concurrent eating, and loss of OH cells produces obesity, suggesting that OH cells facilitate net energy expenditure rather than energy intake [2, 21, 22, 23]. The relationship between OH cells and eating, therefore, remains unclear. Here we investigated this issue at the level of natural physiological activity of OH cells. First, we monitored eating-associated dynamics of OH cells using fiber photometry in free-feeding mice. OH cell activity decreased within milliseconds after eating onset, and remained in a down state during eating. This OH inactivation occurred with foods of diverse tastes and textures, as well as with calorie-free “food,” in both fed and fasted mice, suggesting that it is driven by the act of eating itself. Second, we probed the implications of natural OH cell signals for eating and weight in a new conditional OH cell-knockout model. Complete OH cell inactivation in adult brain induced a hitherto unrecognized overeating phenotype and caused overweight that was preventable by mild dieting. These results support an inhibitory interplay between OH signals and eating, and demonstrate that OH cell activity is rapidly controllable, across nutritional states, by voluntary action.

## Highlights

• Brain orexin cell activity is rapidly inactivated upon the act of eating
• This occurs with different foods, including calorie-free and liquid food
• Complete orexin cell inactivation in adult brain can promote overeating
• Overweight resulting from orexin cell loss is reversible by mild dieting

## Teaser

Brain orexin cells control arousal, but pathological deregulation of their activity can produce sleepiness, obesity, and anxiety. González et al. show that the voluntary act of eating can rapidly downregulate orexin cell activity irrespective of taste or calories, whereas obesity resulting from orexin underactivity can be reversed by a mild diet.

## Results and Discussion

### Natural Population Dynamics of OH Cells during Voluntary Eating

**Figure 1:**
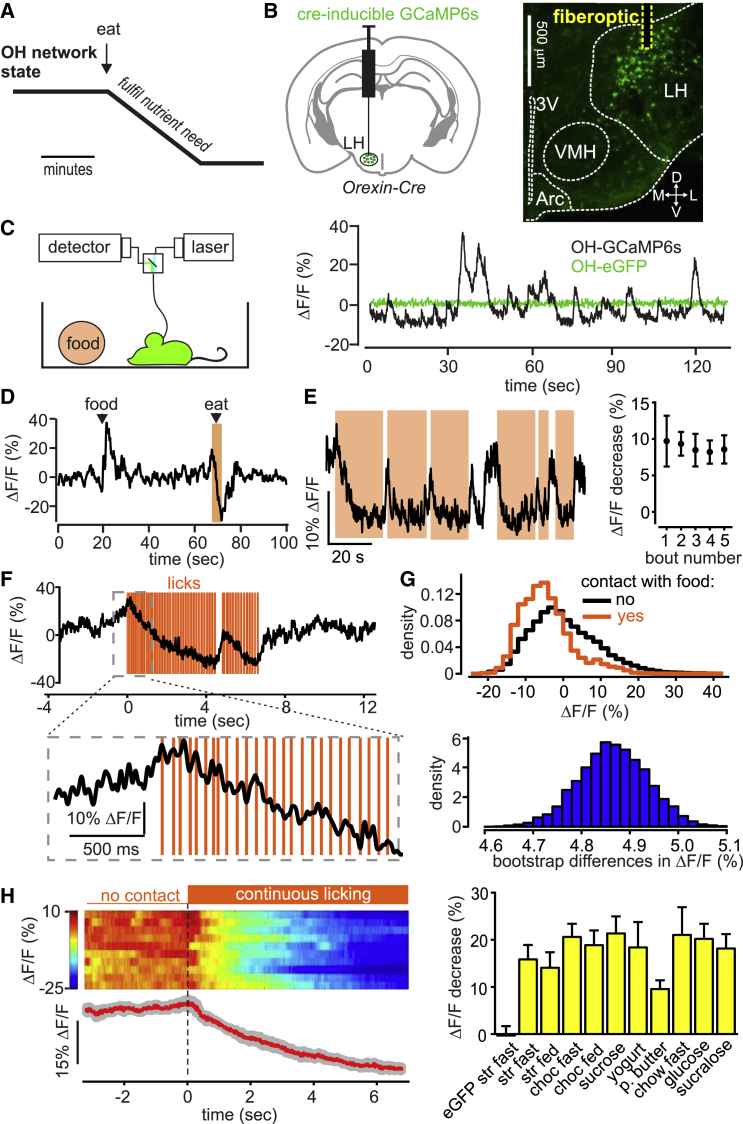
Impact of Eating on Natural OH Cell Dynamics In Vivo (A) A hypothesis for temporal modulation of OH cells during eating. (B) Left: targeting scheme of GCaMP6s to OH cells for obtaining the data shown in this figure (data using alternative targeting of OH cells are shown in Figures S2C–S2F). Right: localization of injection site and path of the optical fiber. 3V, third ventricle; L, D, M, V, lateral, dorsal, medial, ventral; VMH, ventromedial hypothalamus; Arc, arcuate nucleus. Representative image of n = 5 brains. (C) Left: recording scheme. Right: fluorescence trace during cage exploration for mice expressing GCaMP6s or eGFP in OH neurons. Typical examples of n = 5 and n = 3 mice, respectively. (D) Fluorescence trace during introduction of food into the cage and its subsequent consumption (orange-shaded area). Food was a drop of strawberry milkshake. Typical example of n = 5 mice. (E) Left: fluorescence trace during repeated bouts of food contact (orange-shaded areas; food is strawberry milkshake). Typical example of n = 5 mice. Right: quantification of fluorescence change during the first 2 s of consecutive food-contact bouts (means ± SEM, n = 3 mice). (F) Fluorescence change during food licking detected with a touch sensor (food is strawberry milkshake). Typical example of n = 5 mice across eight foods shown in (H), right. (G) Top: probability density of OH cell activity. Bottom: distribution of the bootstrap differences of the same data. Typical example of n = 3 mice. (H) Left: peri-event plots aligned to the onset of licking bouts (dashed line). The heatmap shows individual bouts (two per mouse), and the trace below the heatmap shows the mean of trial averages from each mouse (red line; gray lines represent SEM); n = 5 mice. Right: quantification of the experiment shown on the left, for different foods. Each column shows fluorescence change during the first 4 s of a licking bout (mean signals during 3.5 to 4 s minus signal during −0.5 to 0 s, times relative to the first lick). Data are means ± SEM of n = 4 mice in each group. Left column is control (OH-eGFP mice); other columns are OH-GCaMP6s mice; for food abbreviations, see the Supplemental Experimental Procedures; fast, overnight fasted before the experiment; fed, ad libitum feeding before the experiment. All changes in OH-GCaMP6s mice were significant (p < 0.05 in one-sample t tests of response to each food, DF = 3, t > 3.4). See also Figures S1–S3 and Movie S1.

Orexin/hypocretin (OH) cells are activated by fasting and low glucose levels, and have been hypothesized to drive eating until ingested glucose slowly (within minutes) inactivates them (Figure 1A) [19, 24]. We measured OH cell population activity in freely behaving mice using fiber photometry [17] of the GCaMP6s activity indicator targeted to OH cells while monitoring eating using video tracking or touch sensors (Figure 1B; Figures S1–S3). In freely behaving mice, we observed activity fluctuations in OH-GCaMP6s, but not in OH-eGFP, cells (Figure 1C). The magnitude of these fluctuations (∼10%–40% ΔF/F) was similar to network dynamics recorded with similar methods in other brain regions [25, 26]. Our experimental quantification of photometry performance suggested that >95% of the fluorescence signal would come from ∼0.5 mm from the fiber tip (Figures S2A and S2B), which is well suited to OH cluster dimensions in the mouse hypothalamus. We confirmed that the GCaMP6s signal reflects physiological OH cell modulation by reproducing the previously described in vivo activation of OH cells by sounds [14] and in vitro inhibition of OH cells by glucose [24] (Figures S1C and S1D). The OH-GCaMP6s signal was directly proportional to the OH cell firing rate (Figure S3).

We found that food contact depressed OH cell activity extremely rapidly (<1 s of food contact) (Figures 1D–1H). OH cells returned to an up state within seconds after food contact was stopped (Figures 1D–1F; Figure S2E), suggesting that the rapid OH cell modulation is not caused by slowly varying nutritional signals. This effect was seen in both fasted and fed OH-GCaMP6s mice, but not in the OH-eGFP controls (Figure 1H). For liquid foods, the fall in OH cell activity was apparent within just a few licks (Figure 1F; Figures S2E and S2F). The eating-associated OH cell depression was similar for foods of differing consistency (e.g., chow versus yogurt) and different appetitive value (e.g., chow versus peanut butter) (Figure 1H). To confirm whether caloric content had a role, we tested a zero-calorie “food” (sucralose solution), and still observed robust OH cell inactivation during licking (Figure 1H). Overall, these data show that OH cells are rapidly inactivated by the act of eating, irrespective of food properties or body energy state.

### Natural Impact of OH Neurons on Eating

The above correlative data have two possible causal interpretations: (1) OH cells oppose eating, and are disabled to enable eating, or (2) OH cells drive eating, and so eating stops shortly after OH cells are silenced. To distinguish between these possibilities, we investigated causality between natural OH activity and eating by inactivating OH cells in adult mice through a toxin receptor-mediated cell-knockout strategy [27, 28].

**Figure 2:**
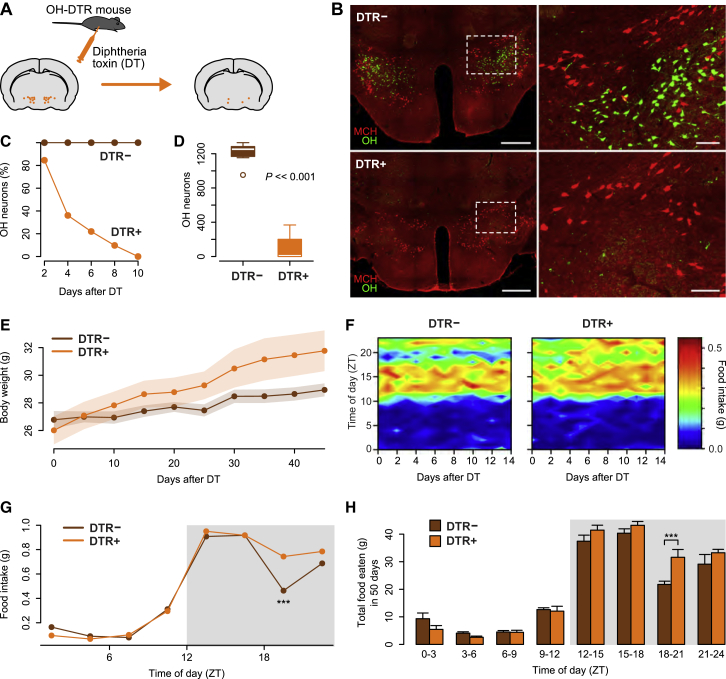
Impact of Natural OH Cell Activity on Spontaneous Feeding Rhythm (A) Strategy for destroying OH neurons in adult mice. (B) Immunostaining for OH (green) and melanin-concentrating hormone-containing (MCH) (red) neurons in DTR^−^ (top) and DTR^+^ (bottom) littermates 10 days after DT injection. The DT injection led to the loss of OH neurons in all brains tested (n = 8). Scale bars represent 500 μm (left) and 100 μm (right). Dashed boxes in the left-hand panels indicate the areas shown in corresponding right-hand panels. (C) Time course of OH cell loss after DT injection in DTR^+^ and their DTR^−^ littermates (n = 5 mice in each group). (D) Quantification of OH cell number >21 days after DT injection in DTR^−^ and DTR^+^ littermates. Unpaired t test, t(13.3) = 16.41, p = 3.2e-10, n = 8 mice in each group. (E) Body weight time series of DTR^−^ and DTR^+^ littermates after DT injection. ANCOVA, F(1, 12) = 12.07, p = 0.005, n = 7 mice in each group. (F) Daily rhythm of eating in DTR^−^ and DTR^+^ mice after DT injection, across days. n = 7 mice in each group. (G) Mean daily rhythm of eating (average of 14 days; gray box is lights off) in DT-injected DTR^−^ and DTR^+^ mice. Repeated-measures ANOVA, interaction: F(7, 84) = 2.38, p = 0.029. Significant differences were found only at the time of day indicated (^∗∗∗^p < 0.001, Holm correction for multiple comparisons). n = 7 mice in each group. (H) Total food consumed after DT injection, relative to the time of day. Repeated-measures ANOVA, interaction: F(7, 84) = 3.07, p = 0.006. Pairwise comparisons revealed statistical differences at the time of day indicated (^∗∗∗^p < 0.001, Holm correction for multiple comparisons). n = 7 animals in each group. See also Figure S4.

We generated new transgenic mice in which the expression of the human diphtheria toxin receptor (DTR) is driven by the OH promoter (see the Supplemental Experimental Procedures). In OH-DTR mice, but not in control WT mice, the injection of diphtheria toxin ablated all OH cells, but not the neighboring melanin-concentrating hormone-containing cells, within a couple of days (Figures 2A–2D). This complete inactivation of OH cells, which is not as readily achievable through alternative silencing methods such as opto- and chemo-genetics, may be critical for elucidation of their full impact, because key deficiency phenotypes are not apparent upon partial inactivation [13].

DT injection led to greater weight gain in DTR^+^ mice than in their DTR^−^ littermates (Figure 2E), confirming that OH cells oppose overweight. Next, we probed food intake patterns at an hourly temporal resolution, using a food hopper specifically designed to re-capture any food spillage and a food-weighing system whose errors were sufficiently low to report changes greater than 0.01 g (Figures S4A–S4D). In DTR^−^ mice injected with DT (control mice) this revealed a robust daily eating rhythm, where food intake was largely restricted to the lights-off phase (“night”) but had a pronounced “dip” late at night (Figures 2F and 2G). However, in DTR^+^ mice injected with DT, this dip in eating was significantly reduced, causing them to consume significantly more food during the late night (Figures 2F–2H; note that the magnitude of this overeating is well above the sensitivity limit of the food-weight detector; Figures S4A–S4D). Interestingly, this overeating did not cause compensatory undereating at other times of day (Figures 2F–2H). Glucose tolerance in OH cell-deficient mice was normal (Figure S4E), as previously observed for OH-deficient humans [29], suggesting that OH cell loss does not prevent glucose uptake from blood into tissues. Overall, these data show that the natural OH cell activity prevents overeating and suppresses weight gain.

### Temporal Dissociation of Fasting-Dependent and OH-Dependent Eating

**Figure 3:**
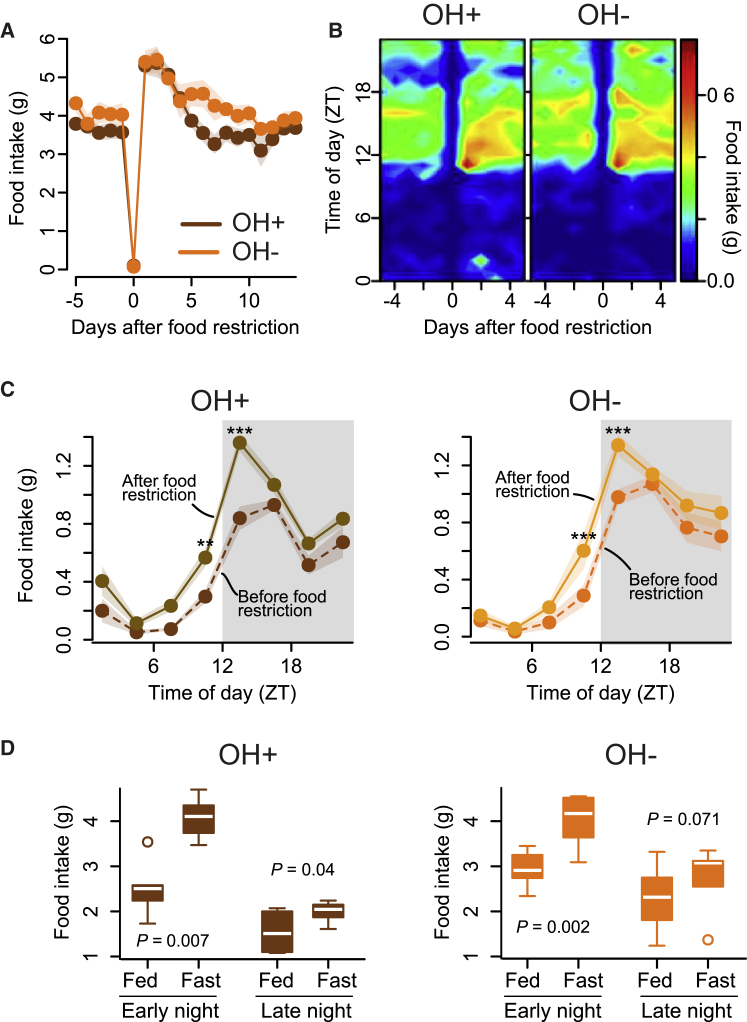
Impact of Natural OH Cell Activity on Rebound Eating after Fasting (A) Eating responses to a 1-day fast in control mice (OH^+^, DTR^−^ mice injected with DT) and their OH cell-deficient littermates (OH^−^, DTR^+^ mice injected with DT). n = 6 mice in each group. (B) Daily eating rhythms before and after a 1-day fast in OH^+^ and OH^−^ littermates. n = 6 mice in each group. (C) Daily eating rhythms 3 days before and after food restriction. Repeated-measures ANOVA, interaction: F(7, 35) = 3.36, p = 0.008 (left) and F(7, 35) = 3.53, p = 0.006 (right). Follow-up tests showed significant differences only at times marked with asterisks (^∗∗^p < 0.01, ^∗∗∗^p < 0.001, Holm correction for multiple comparisons). n = 6 mice in each group. (D) Total food consumed 3 days before (fed) and 3 days after (fast) food restriction in OH^+^ and OH^−^ mice (n = 6 in each group) during early night (ZT12–14) compared to that consumed during late night (ZT18–20). Paired t tests.

The above findings demonstrate that the natural activity of OH cells opposes eating, and therefore challenge current models postulating that OH cells stimulate eating. However, because OH cells are activated by fasting, it is still possible that they become critical for compensatory eating after fasting [11]. To examine this possibility, we measured rebound overeating after fasting in OH^−^ mice (i.e., DTR^+^ mice injected with DT), but surprisingly found it similar to their OH^+^ littermates (DTR^−^ mice injected with DT) (Figures 3A and 3B). Furthermore, although both the OH^−^ and OH^+^ mice overate for several days after fasting, on each of these days they ate more only in the early-night phase (Figures 3C and 3D), i.e., a different phase from that when food intake was naturally regulated by OH cells (Figure 2G). This temporal dissociation reveals that, at least under the conditions studied here, nutrient shortage and OH cells regulate distinct daily phases of food intake. These data suggest that OH cells are not required for compensatory overeating after fasting, at least when food is readily available.

### Normalizing Eating Prevents Weight Gain in OH Cell-Deficient Mice

Identifying an effective strategy for body weight control in OH-deficient individuals is of clinical interest [21, 30]. In our experiments, the overeating in the OH^−^ mice (∼10 g of food over 50 days or ∼2% daily) could be theoretically sufficient to account for the overweight in these mice based on the following reasoning. The OH^−^ mice gain ∼10% weight (relative to OH^+^ controls) over about 45 days, i.e., ∼0.2% daily excess weight gain (Figure 2E). Our fasting experiment shows that, in 1 day, a 100% change in food intake can cause a 10% change in weight (Figure 3A). If this 10:1 relation holds in general, then the ∼2% daily eating increase would be sufficient to account for the 0.2% daily excess weight gain.

**Figure 4:**
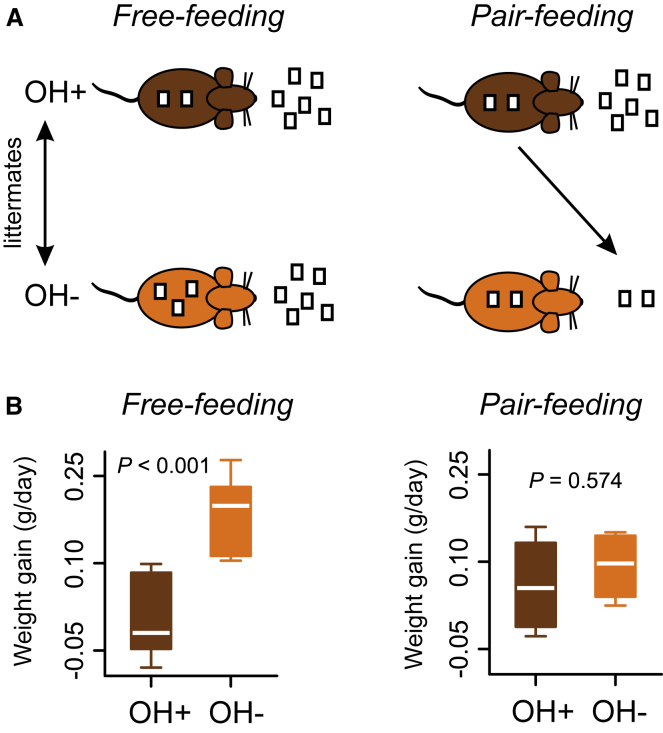
Prevention of Weight Gain Caused by OH Cell Loss by Dieting (A) Strategy for pair-feeding experiment (OH^+^, DTR^−^ mice injected with DT; OH^−^, their DTR^+^ littermates injected with DT). (B) Weight gain of OH^−^ and OH^+^ littermates during weeks 2 and 3 after DT injection, and during free feeding (unpaired t test, t(11.93) = −4.327, p = 0.0009, n = 7 mice in each group) and pair feeding (unpaired t test, t(5.41) = −0.598, p = 0.574, n = 4 animals in each group).

Therefore, we investigated whether the excess weight gain in the OH^−^ mice can be controlled by mild dietary restriction. To achieve this, we employed the pair-feeding paradigm [31], where the food available to each OH^−^ mouse was matched daily to that eaten by its OH^+^ littermate control (Figure 4A). This effectively fixes food intake, but in a mild physiological manner without abnormal periods of fasting [31].

Pair feeding the OH^−^ mice prevented them from becoming heavier than their OH^+^ siblings (Figure 4B). In contrast, in control experiments performed during the same time, free-feeding OH^−^ mice became significantly heavier than their OH^+^ siblings (Figure 4B). Note that this does not exclude that factors other than eating may mediate weight gain of OH^−^ mice, for example, alterations in sleep/wake rhythms and energy expenditure [2, 31, 32]. However, our results do suggest that, in the absence of overeating, such factors are insufficient to cause the excessive weight gain.

### Conclusions

Our findings reveal population activity dynamics of OH cells during eating, and suggest that eating is less likely to occur when OH cells are naturally active. OH cells stimulate “non-eating” behaviors such as locomotion [18]. Although these behaviors expend energy, it is difficult to eat while performing them. Thus, a possible evolutionary rationale for our findings is that suppression of OH-dependent non-eating behaviors—by silencing OH cells upon food contact—would facilitate eating (e.g., by making the mouse less likely to move away from the food). It is tempting to speculate that increased eating after experimental stimulation of the OH system [11, 13] may be a secondary response to OH-driven energy expenditure [33], rather than a primary function of OH cells.

Our findings identify interesting directions for future work. We found that the weight gain in OH-cell-deficient mice could be prevented by mild caloric restriction. This suggests that development of non-pharmacological interventions may be useful in managing excessive weight gain in neuropsychiatric conditions associated with reduced OH signaling [3, 21, 30, 34]. Furthermore, our results suggest that OH cells—whose hyperactivity has been implicated in pathological states such as panic anxiety [5]—could be inhibited by simple voluntary actions such as eating, irrespective of the nature of the food. Further research into neuroscience-based lifestyle interventions for anxiety and obesity might lead to treatments that are easier to implement and have fewer side effects.

## Experimental Procedures

OH cell activity was recorded in vivo using fiber photometry of the GCaMP6s activity indicator targeted to OH cells either using previously characterized orexin-Cre mice and Cre-inducible GCaMP6s viral vectors [35, 36] (Figure 1; Figure S1B) or using a newly generated orexin promoter-dependent adeno-associated virus (AAV)-GCaMP6s vector (characterized in Figures S2C–S2F and described in the Supplemental Experimental Procedures). OH cells were specifically and completely inactivated using a diphtheria toxin receptor-mediated cell-ablation strategy in newly generated OH-DTR transgenic mice described in the Supplemental Experimental Procedures. Food intake was monitored using a TSE PhenoMaster system, whose sensitivity and accuracy were directly determined in our laboratory (Figures S4A–S4D). Immunohistochemistry and glucose tolerance tests were performed using standard techniques (see the Supplemental Experimental Procedures).

## Author Contributions

J.A.G. conducted most of the experiments; L.T.J. created and characterized the DTR transgenic mice; P.I. conducted the experiments in Figures S2C–S2F; M.S. contributed to the experiments in Figure S2C; D.B. and L.F. designed the study and obtained funding; and D.B., L.F., and L.T.J. wrote the paper.

## Supplemental Information

Document S1. Supplemental Experimental Procedures and Figures S1–S4

Movie S1. Real-Time Recording of Orexin/Hypocretin Population Activity, Top Left, during a Continuous Licking Bout in a Freely Moving Mouse, Related to Figure 1 The arrow shows the location of the food spout containing liquid food (strawberry milkshake). The red dot marks the time when the mouse starts licking.

Document S2. Article plus Supplemental Information
