# Supplementary material for: Inhibitory Interplay between Orexin Neurons and Eating
Source: Curr Biol. 2016 Sep 26;26(18):2486–91. doi: 10.1016/j.cub.2016.07.013 (PMC5049542; doi:10.1016/j.cub.2016.07.013)
Supplement: Document S1. Supplemental Experimental Procedures and Figures S1–S4 [file mmc1.pdf]

Current Biology, Volume *26*

Supplemental Information

Inhibitory Interplay between

Orexin Neurons and Eating

J. Antonio González, Lise T. Jensen, Panagiota Iordanidou, Molly Strom, Lars Fugger, and Denis Burdakov

Supplemental Figure 1

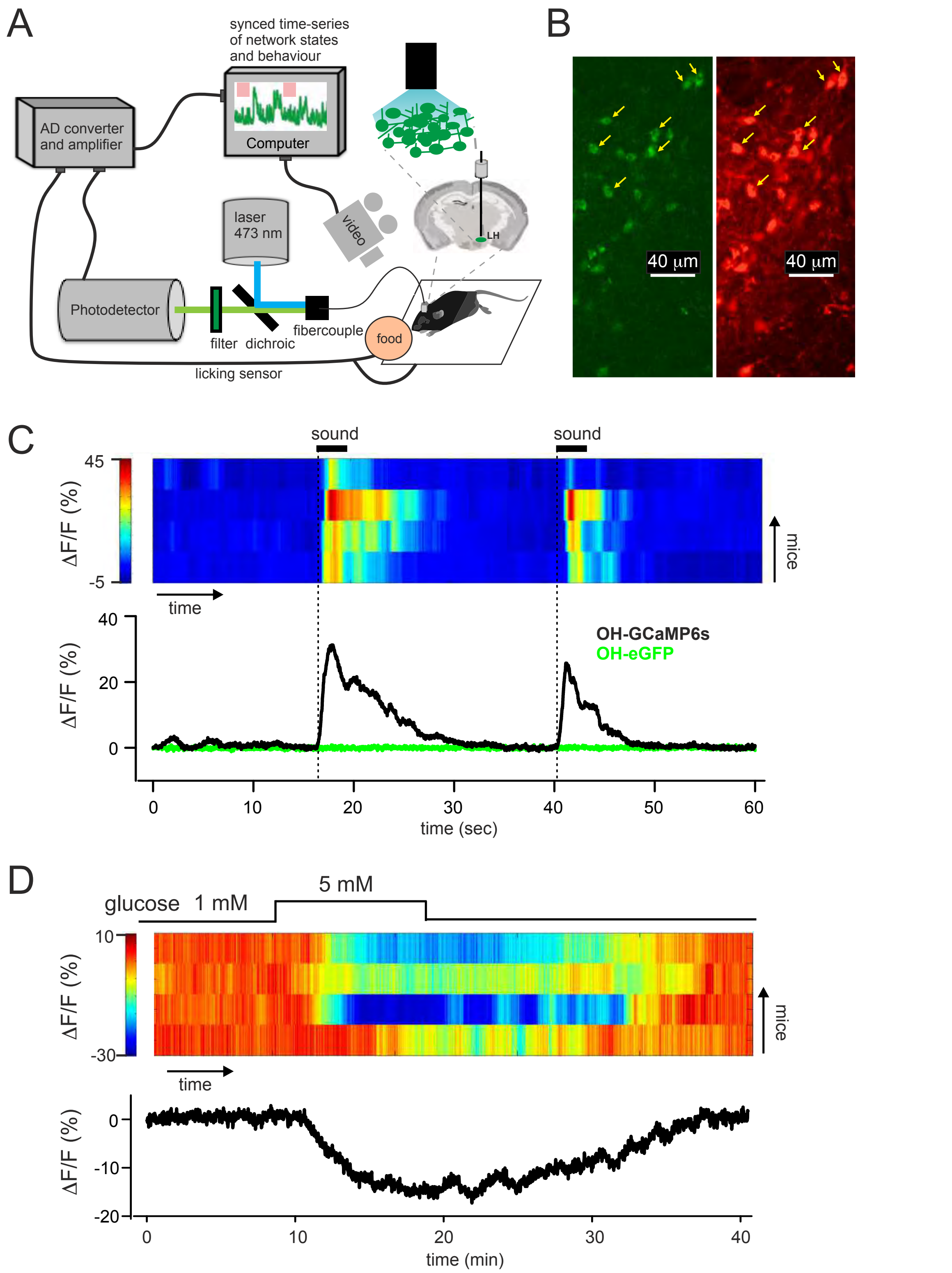

Supplemental Figure S1 (related to Figure 1)

A. Detailed scheme for co-registering neural activity and eating.

B. OH-GCaMP6s neurons (left, green) immunostained for OH (right, red) in an LH brain slice; some of the corresponding cells are arrowed.

C. Fiber photometry recording of fluorescence responses of freely-moving mice to a sudden novel sound (16 kHz, 100 db), in OH-GCaMP6s mice (n = 4, single trials from individual mice shown on heat-map, averaged responses shown below heat-map), and in OH-eGFP mice (n = 3 mice, black trace is averaged response).

D. Fiber photometry recordings of fluorescence responses in acutely isolated brain slices of OH-GCaMP6s cells to changes in ambient glucose concentration. Brain slices were prepared, and extracellular glucose level was manipulated, as in reference S3. The fiber tip was placed 100-300 µm above OH-GCaMP6s neurons in the lateral hypothalamus. Heat-map shows individual trials, plot below the heat map shows average of the trials.

Supplemental Figure 2

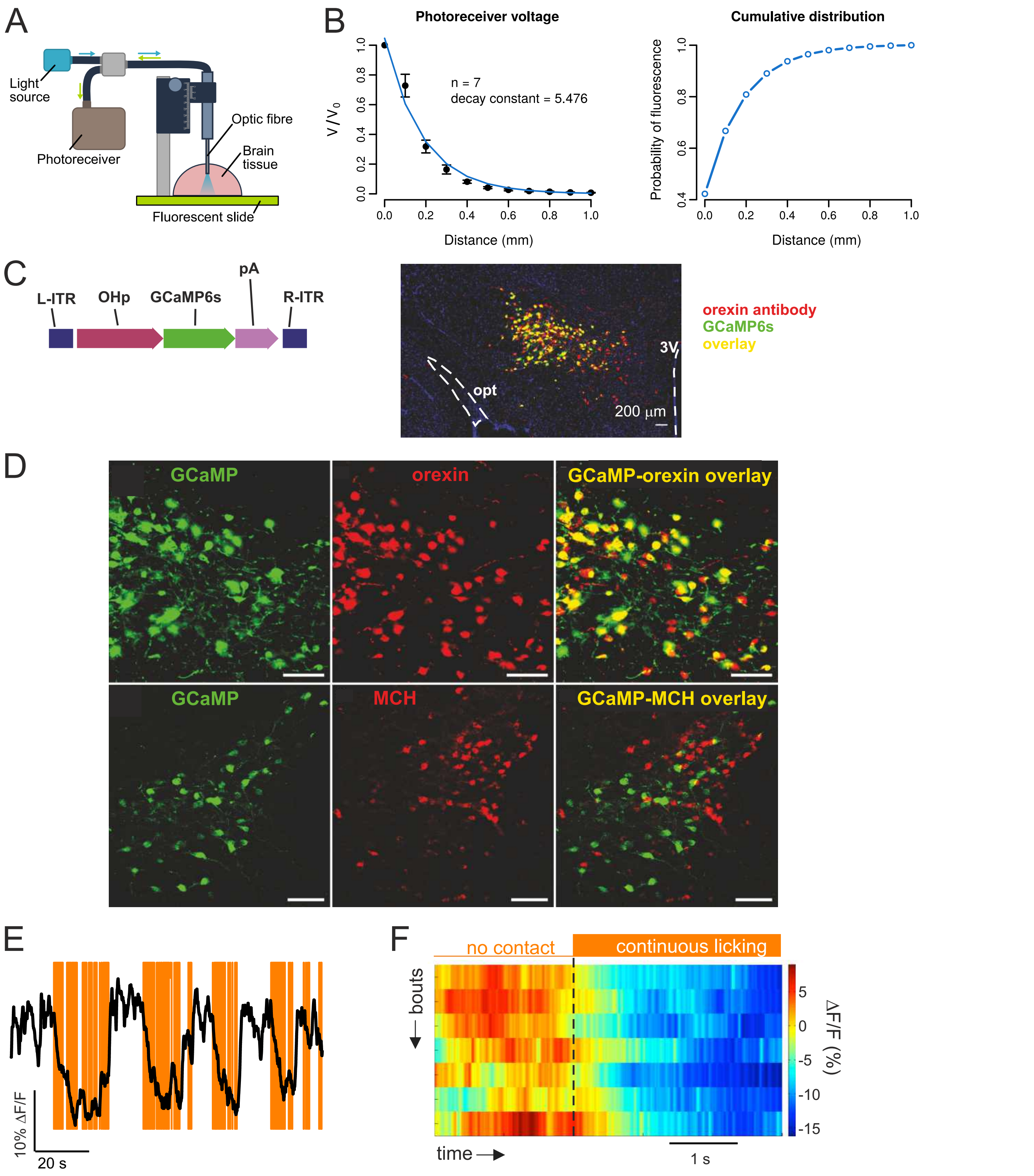

Supplemental Figure S2 (related to Figure 1). Characterization of fluorescence capture by the photometry fiber, and experiments using GCaMP6s expressed under control of the preproorexin promoter.

A. Scheme of experimental set-up. To characterize light capture from fluorescent objects in brain tissue, we measured fluorescence from a slide with an optic fiber placed at increasingly greater distances. The slide had similar fluorescence emission to GCaMP6.

B. *Left*, normalized fluorescence signal detected at different distances from the fiber tip. *Right*, Cumulative probability of fluorescence as a function of distance from the tip (calculated from data on the left). This shows that > 95% of fluorescence comes from within 0.5 mm depth from the fiber tip, which corresponds to a volume of 0.048 mm^3 (volume calculated as a conical frustum for a fiber of NA 0.37 and 0.2 mm diameter, and brain refractive index of 1.369 based on Sun et al., 2012, Opt Express 20: 1084-1095).

C. *Left,* Schematic of AAV construct. L- and R- ITR, left- and right- inverted terminal repeat; OHp, preproorexin promoter; pA, human growth hormone polyA. *Right*, Immunohistochemistry of the lateral hypothalamus (LH) of a WT C57BL6 mouse injected with the AAV2/1-OH-GCaMP6s virus. Orexin (Santa Cruz goat anti-orexin-A, 1:2000) labelling is in red and GCaMP6s in green. DAPI is shown in blue. Opt, optic tract; V3, third ventricle.

D. *Top row*, Same as C (right) at higher zoom: orexin immunoreactivity was detectable in 96.3% (1779/1848 cells from 3 brains) of GCaMP6s neurons. *Bottom row,* Melanin concentrating hormone (Phoenix pharmaceuticals, rabbit anti-MCH, 1:2000) labelling in a mouse injected with AAV2/1-OH-GCaMP6s virus. No co-localization of GCaMP6s and MCH immunoreactivity was observed. Scale bars, 100 µm.

E. Fiber photometry of OH-GCaMP6s fluorescence signal (black) during strawberry milkshake licking (orange). Experimental set-up is the same as in Fig. 1F, except GCaMP6s targeting was performed as in panels A-B above. Typical example of n = 4 mice.

F. Peri-event plots aligned to onset of licking bouts (dashed line). Heatmap shows consequitive licking bouts from the same mouse. Experimental set-up is the same as in E. Typical example of n = 4 mice.

Supplemental Figure 3

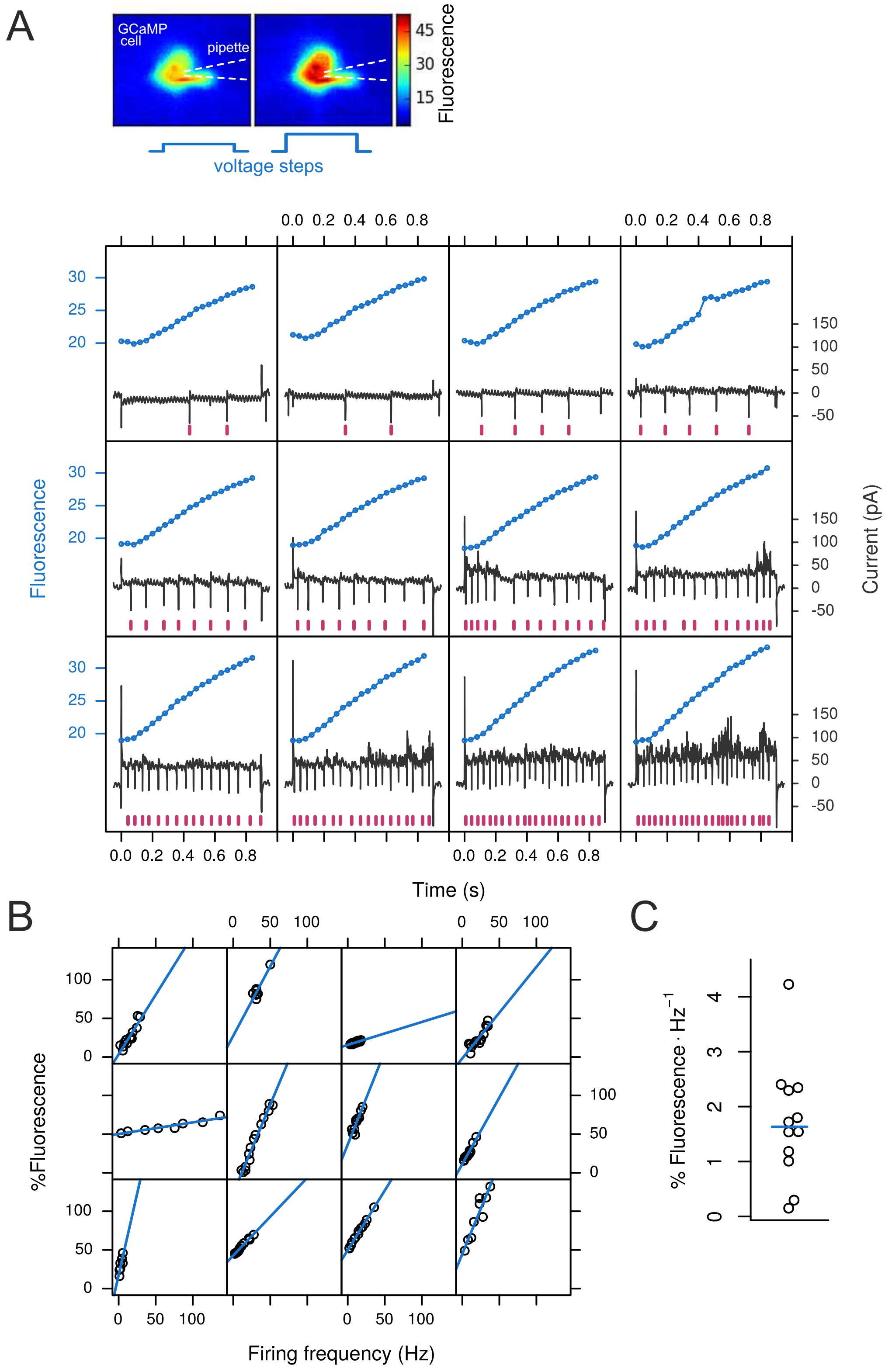

Supplemental Figure S3 (related to Figure 1).
GCaMP6s fluorescence changes associated with OH-GCaMP6s cell firing changes.

A. Top, raw images from the experiment. Bottom, examples of GCaMP6s recordings at different firing states (method described in Supplementary Methods, Section 1). Fluorescence is reported in raw pixel values (blue) and action potentials are detected (magenta) from the raw electrophysiology traces (black). Typical example of n = 12 cells.

B. Examples of the relationship between GCaMP6s signal and action potential firing in 12 different OH-GCaMP6s cells. Percent fluorescence was calculated from raw signals, such as those shown in A, as dF/F0 x 100, where F0 is the fluorescence at the beginning of the depolarizing pulse and dF is the difference in fluorescence between the end and the beginning of that same pulse.

C. Change in GCaMP6s fluorescence per Hz of firing rate for 12 OH-GCaMP6s cells, obtained from the slope of the linear fit of data in B. The median value (blue line) represents 1.6% fluorescence change for every 1 Hz firing change.

Supplemental Figure 4

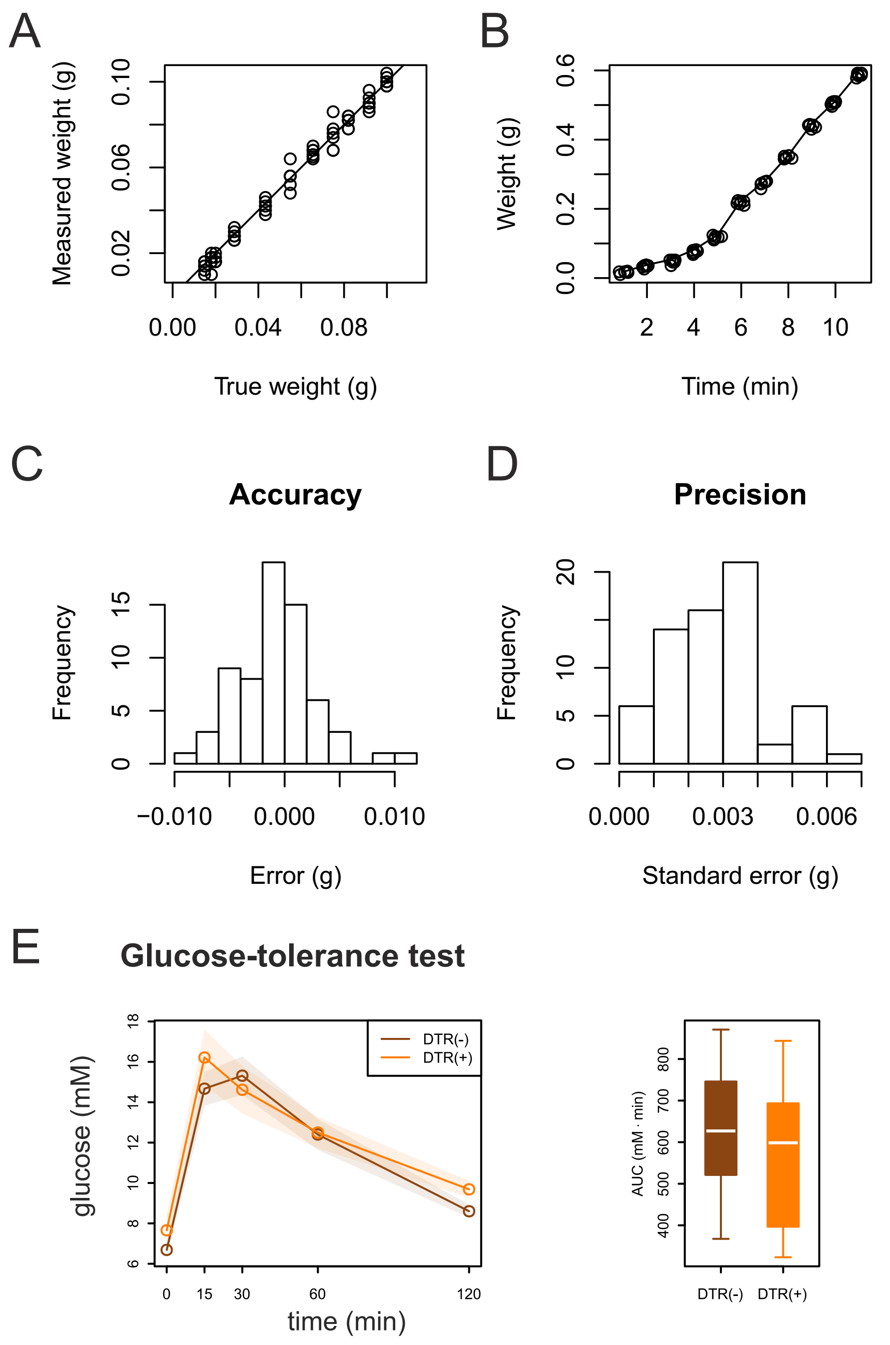

Supplemental Figure S4 (Related to Figure 2)
Determination of feeding sensor sensitivity. Each sensor was calibrated as usual and the food container was filled up. Then, a set of weights (n = 11, range 15 to 100 mg) were removed in sequence from the food container to simulate food intake. This test was repeated 5 times for each of 6 feeding sensors for a total of 330 measurements.

A. Relationship between true weight (line) and measured weight (points).
B. Temporal relationship between cumulative true weight (line) and measured weight (points), showing that weight can be accurately recorded on the time-scale of minutes.
C. Accuracy, illustrated as the frequency distribution of errors (differences between true and measured weight). The system is accurate to 0.01g.
D. Precision (a measure of repeatability), representing how close measurements made under the same conditions are to each other. The mean standard error is below 0.008 g.
E. Glucose tolerance analysis in mice with OH cells, DTR(-), and mice without OH cells, DTR(+); data are means and SEM of n = 7 mice (see Supplementary Methods).

SUPPLEMENTAL EXPERIMENTAL PROCEDURES

Targeting activity indicators to orexin/hypocretin neurons

Animal procedures were performed following UK Home Office regulations, and adult male mice were used in all experiments.

For calcium imaging, activity indicator GCaMP6s was targeted to OH neurons using two alternative methods. In Method 1 (Fig. 1, Fig. S1), AAVs carrying Cre-dependent GCaMP6s (rAAV9.CAG.Flex.GCaMP6s.WPRE.SV40; Penn Vector Core) or eGFP (AAV1.CAG.Flex.eGFP; UNC Vector Core) were stereotaxically injected into LH of orexin-Cre transgenic mice [S1, S2]. The specificity of Cre targeting to OH cells in this orexin-Cre line was quantified and described elsewhere [S1], and confirmed here (Fig. S1B). Cre-dependency of viral expression was confirmed by injections of the Cre-dependent viruses into the brains of Cre-negative C57BL6 mice (n = 3-6 mice for each virus).

Alternatively (Method 2, illustrated in Fig. S2E-F), GCaMP6s was targeted to OH neurons of WT C57BL6 mice using AAV vector that expresses GCaMP6s under the control of the preproorexin promoter (kind gift of Takeshi Sakurai). This promoter has been previously shown to target OH cells with high fidelity [S3], which is confirmed here (Fig. S2C-D). To make this AAV vector, we amplified the preproorexin promoter using oligonucleotides 5'–GTTCCTGCGGCCGCACGCGTGAGCTCAATAAAGAGGTT–3' and 5'–GGCAAGCTTCTGCAGGTCGACGGTGTCTGGCGC – 3', and cloned it into the MluI and SalI sites of pAAV-MCS (Agilent Technologies) using In-Fusion HD Cloning kit (Clontech), to create pAAV-OH. GCaMP6s gene was amplified from plasmid pGP-CMV-GCaMP6s (Addgene Plasmid #40753; [S4]) using oligonucleotides: 5'–TGAGCGCCAGACACCGTCGACCATGGGTTCTCATCAT–3' and 5'– GGGATGCCACCCGTAGATCTTCACTTCGCTGTCATCAT – 3' and cloned into pAAV-OH vector SalI and BglII sites as above to create pAAV-OH-GCaMP6s. Sequences of preproorexin promoter and GCamp6s were verified by sequencing using several internal oligonucleotides. AAV virus was prepared as described in [S5].

Three 50 nl injections of the Cre-dependent or the preproorexin promoter -dependent GCamp6s virus were made into orexin-Cre or WT C57BL6 mice respectively, at the following coordinates: 1.35 mm caudal from bregma; ±0.9 mm lateral from midline; and 5.30, 5.20, and 5.10 mm ventral from brain surface. A fiberoptic implant was stereotaxically installed with the fiber tip above the lateral hypothalamus, and fixed to the skull as previously described [S6].

In Fig. S3, to investigate the relationship between GCaMP6s signals and OH cell firing, we used cell-attached electrophysiology together with epifluorescence recording, in acute mouse brain slices. OH-Cre cells were tagged with GCaMP6s as described above, and their activity was measured using the cell-attached recording in voltage-clamp mode [S7]. Depolarizing voltage steps were applied to the cell, and GCaMP6s signals were elicited using a xenon excitation lamp and standard eGFP filters, and captured at 25 fps using a DAGE-MTI camera.

Fiber photometry

A fiber photometry setup was built according to general principles outlined in Cui et al., 2013, except a single multimode fiber was used for excitation and emission inside the brain as in [S8]. Specifically, the excitation light was sent into a fiber-coupled cube containing a dichroic mirror and GFP filters (Doric, FMC_GFP_FC), and from there into a patchchord (Doric, MFP_200/230/900-0.22_2m_FCM-MF) plugged into the fiberoptic implant (Doric, MFC_200/260-0.37_50mm_MF2.5(7.5mm)_FLT) using a brass connector (Doric, SLEEVE_BR_2.5). A 473 nm laser (Becker & Hickl) provided the excitation light. The laser power at the implant fiber tip was measured before implantation (X-Cite Optical Power Measurement System, Excelitas Technologies) and adjusted to 0.1 mW. Emitted fluorescence was fiber-coupled from the GFP cube to a photodetector (Becker & Hickl, HPM-CON-2). The analogue detector signal was sent to an AD port of a HEKA EPC-10 amplifier. The fluorescence signal was recorded using software provided with the amplifier (HEKA Patchmaster). Photometry data underwent minimal processing, consisting of standard within-trial normalization, in which the signal $\Delta F/F$ was defined as $100*(F-F_{mean}) / F_{mean} - 100$, where F is the raw fluorescence signal and F_{mean} is the mean fluorescence intensity of a 10 min baseline period before trial. Fiber tips were implanted at the following coordinates: 1.38 mm caudal from bregma; 0.95 mm lateral from midline; and 5 mm ventral from brain surface. The LH location of fiber tips were verified post-recording by examining slices with visible fiber tract; because most of these slices were damaged, intact slices closest to the fiber tip in the rostrocaudal plane were used for Fig. 1B, and the fiber tract was drawn.

Eating behavior quantification

Animals were kept on a 12h light-dark cycle (lights on at 7am). To control for circadian factors, the experiments were performed either during the dark phase (9pm-11pm) or the light phase (12pm-5pm); no differences in the described responses were observed between these circadian phases. Eating was defined as food-mouth touch, and its occurrence was precisely quantified by a custom touch sensor for wet food, built according to previously described specifications [S9]. The temporal relation between fluorescence and touch was preserved by collecting and processing the two signals simultaneously on the same AD board. Occasionally, the time of food contact was estimated from video-recordings time-synced to the photometry trace (Fig. 1E, Supplemental Movie 1). Foods examined in experiments shown in Fig. 1H were: str = strawberry milkshake (Yazoo), choc = chocolate milkshake (Yazoo), p.butter = peanut butter (Essential Waitrose), chow = wet crushed standard mouse chow, sucrose (0.4 M), sucralose (1.5 mM), yogurt (Yeo Valley plain), glucose (0.3 M). The examples shown in Fig. 1F, 1H (left) are from strawberry milkshake. Mice were habituated to all foods to remove effects of novelty.

Generation of orexin-DTR transgenic mice

As mice are insensitive to diphtheria toxin (DT), we generated transgenic mice expressing the human diphtheria toxin receptor driven (DTR) by the OH promoter in order to perform selective killing of OH neurons upon DT administration. The orexin promoter was obtained from a previously published construct [S3]. We exchanged the GFP cDNA fragment with a 632 bp HB-EGFP cDNA SalI-NotI fragment amplified from orfeome clone HAIB:100067676 (Geneservice). A 3.3 kb XhoI-BfrI fragment was excised and microinjected into pronuclei from B6D2 mice by standard methods. Transgenic founder mice were identified by PCR. Expression of functional DT receptors was validated by injection of DT followed by counting of immunoreactive OH neurons. Complete absence of OH neurons in the hypothalami of OH-DTR mice treated with DT confirmed transgenic expression in all of the OH cells. Following breeding and characterization we picked one of two phenotypically similar founder lines for all subsequent studies.

Food intake monitoring and pair-feeding

Adult male mice were housed individually in metabolic cages equipped with automated anti-spill food-weighing devices (TSE PhenoMaster), and allowed to acclimatise for one week before the experiments, during which eating was measured and confirmed to reach a steady state. In the food hopper, the food could only be eaten by standing on a grating below the food source; any food spillage was thus captured by the grating and not counted as food intake. We have performed experiments to determine the TSE PhenoMaster sensitivity in our experimental room (since the sensitivity can depend on room vibration), which demonstrated that the system is accurate to 0.01g with a mean standard error of less than 0.008 g (Fig. S4A-D). This directly confirms that the food intake changes we discovered (≈0.2 g) are well above the range of measurement error. Diet was standard chow (LabDiet 5021). Animals were kept on a 12h light-dark cycle (lights on at 7am). Room temperature was 22°C. In circadian analysis of behaviour, the time is given as 24h Zeitgeber (ZT) time, where ZT0 corresponds to lights on time, and ZT12 corresponds to lights off. Body weight was measured manually on a daily basis at the end of the lights-on period (between ZT10–ZT11). Diphtheria toxin (DT) (Sigma D0564) was diluted to 1 μg/ml and injected i.p.; two DT doses (at days 0 and 2) of 100 ng each were given. The animals were between 7 and 12 weeks old on the day of DT injection.

The extent to which excessive body weight gain is due to increased food intake was investigated using a previously described pair-feeding paradigm [S10]. In the pair-feeding experiments, the animals were individually housed in metabolic cages and injected with DT as described above. The pair-feeding protocol was started one week after DT injection because it takes several days for DT to have an effect on OH cell count (Fig. 2C). The DTR(-) mice had free access to food and their intake was measured automatically (PhenoMaster). The amount of food consumed by each of the DTR(-) animals during a 24-h period was then given its DTR(+) littermate during the following 24 hours. For food restriction experiments (Fig. 3), the food was removed for 24 hours at the end of the light period (ZT10–ZT11). Water access was not restricted.

Histology

The mice were perfused with PBS followed by 4% PFA, and the brains were removed and placed in 30% sucrose in PBS for cryopreservation. The brains were then frozen on dry ice with OCT compound and stored at −80°C until needed. 30 μm-thick coronal sections of the lateral hypothalamus were cut with a cryostat (Leica CM3050 S). The sections were stored in cryoprotectant at −20°C until needed.

Of these, every sixth section was taken for immunohistochemistry, which was performed with overnight incubation with a goat anti-orexin primary antibody (1:2000, Santa Cruz Biotechnology sc-8070), followed by a 2 hr incubation with anti-goat Alexa Fluor 488 (1:400; Invitrogen A11055) secondary antibody. To confirm the presence of MCH neurons we used a rabbit anti-MCH primary antibody (1:2000; Phoenix Pharmaceuticals H-070-47) and an anti-rabbit Alexa 568 secondary (1:400; Invitrogen A11011). Hoechst was used for nuclear counter-staining. Full-section images were captured semi-automatically with an Olympus virtual slide scanning micro- scope (VS120, 10x objective). Fiji open source software was used for image processing and the cells were counted manually using the PointPicker plugin.

Glucose tolerance tests

Intraperitoneal glucose tolerance tests were performed in DTR(+) and DTR(-) littermates after an overnight fasting period (~18h fast). A 25% aqueous glucose solution was administered by i.p. injection at a dose of 1g glucose per kg weight [S11]. Blood samples were taken from the tip of the tail 0, 15, 30, 60 and 120 min later (Fig. S4E), and glucose was measured with a blood glucose monitor (Accu-Chek Aviva Nano, Roche Diagnostics). The area under the curve (AUC; Fig. S4E) above baseline was calculated using the trapezoid rule; an unpaired *t* test was used to test for statistical significance.

Data analysis

Data were analyzed and plotted with R 3.2.0 (R Core Team, 2015), Prism 6 (GraphPad software), or Origin 2015 (Microcal). Statistical significance was tested as described in the figure legends, and $p < 0.05$ was considered significant. We used bootstrap methods [S12] to test the hypothesis that licking correlates with changes in GCaMP6s signals (Fig 1G). Values of fluorescence obtained during licking were randomly sampled (with replacement) and their mean was calculated. The same was done with samples obtained during non-licking, and this procedure was repeated 10000 for each of the two data sets. The distribution of bootstrap means is illustrated in Fig 1G, top, whereas Fig 1G, bottom, shows the distribution of the differences of the bootstrap mean values. In all cases these differences were above 0 (for Fig 1G, bottom, the mean difference was 4.87% and 95% confidence interval was [4.75, 4.98]), showing that GCaMP6s fluorescence increased with licking and that this increase was unlikely to occur by chance.

SUPPLEMENTAL REFERENCES

S1. Schöne, C., Cao, Z.F., Apergis-Schoute, J., Adamantidis, A., Sakurai, T., and Burdakov, D. (2012). Optogenetic probing of fast glutamatergic transmission from hypocretin/orexin to histamine neurons in situ. J. Neurosci. *32*, 12437-12443.

S2. Matsuki, T., Nomiyama, M., Takahira, H., Hirashima, N., Kunita, S., Takahashi, S., Yagami, K., Kilduff, T.S., Bettler, B., Yanagisawa, M., et al. (2009). Selective loss of GABA(B) receptors in orexin-producing neurons results in disrupted sleep/wakefulness architecture. Proc. Natl. Acad. Sci. U S A *106*, 4459-4464.

S3. Burdakov, D., Jensen, L.T., Alexopoulos, H., Williams, R.H., Fearon, I.M., O'Kelly, I., Gerasimenko, O., Fugger, L., and Verkhratsky, A. (2006). Tandem-pore K+ channels mediate inhibition of orexin neurons by glucose. Neuron *50*, 711-722.

S4. Chen, T.W., Wardill, T.J., Sun, Y., Pulver, S.R., Renninger, S.L., Baohan, A., Schreiter, E.R., Kerr, R.A., Orger, M.B., Jayaraman, V., et al. (2013). Ultrasensitive fluorescent proteins for imaging neuronal activity. Nature *499*, 295-300.

S5. Velez-Fort, M., Rousseau, C.V., Niedworok, C.J., Wickersham, I.R., Rancz, E.A., Brown, A.P., Strom, M., and Margrie, T.W. (2014). The stimulus selectivity and connectivity of layer six principal cells reveals cortical microcircuits underlying visual processing. Neuron *83*, 1431-1443.

S6. Jego, S., Glasgow, S.D., Herrera, C.G., Ekstrand, M., Reed, S.J., Boyce, R., Friedman, J., Burdakov, D., and Adamantidis, A.R. (2013). Optogenetic identification of a rapid eye movement sleep modulatory circuit in the hypothalamus. Nat. Neurosci. *16*, 1637-1643.

S7. Perkins, K.L. (2006). Cell-attached voltage-clamp and current-clamp recording and stimulation techniques in brain slices. J. Neurosci. Methods *154*, 1-18.

S8. Gunaydin, L.A., Grosenick, L., Finkelstein, J.C., Kauvar, I.V., Fenno, L.E., Adhikari, A., Lammel, S., Mirzabekov, J.J., Airan, R.D., Zalocusky, K.A., et al. (2014). Natural neural projection dynamics underlying social behavior. Cell *157*, 1535-1551.

S9. Hayar, A., Bryant, J.L., Boughter, J.D., and Heck, D.H. (2006). A low-cost solution to measure mouse licking in an electrophysiological setup with a standard analog-to-digital converter. J. Neurosci. Methods *153*, 203-207.

S10. Ellacott, K.L., Morton, G.J., Woods, S.C., Tso, P., and Schwartz, M.W. (2010). Assessment of feeding behavior in laboratory mice. Cell. Metab. *12*, 10-17.

S11. Ayala, J.E., Samuel, V.T., Morton, G.J., Obici, S., Croniger, C.M., Shulman, G.I., Wasserman, D.H., McGuinness, O.P., and Consortium, N.I.H.M.M.P.C. (2010). Standard operating procedures for describing and performing metabolic tests of glucose homeostasis in mice. Dis. Model. Mech. *3*, 525-534.

S12. Calmettes, G., Drummond, G.B., and Vowler, S.L. (2012). Making do with what we have: use your bootstraps. J. Physiol. *590*, 3403-3406.

Legend of Supplemental Video (related to Figure 1)

Real-time recording of orexin/hypocretin population activity (top left) during a continuous licking bout in a freely-moving mouse. Arrow shows the location of food spout containing liquid food (strawberry milkshake). Red dot marks the time when the mouse starts licking.
