# Supplementary figures and images for: Inhibitory Interplay between Orexin Neurons and Eating
Source: Curr Biol. 2016 Sep 26;26(18):2486–91. doi: 10.1016/j.cub.2016.07.013 (PMC5049542; doi:10.1016/j.cub.2016.07.013)

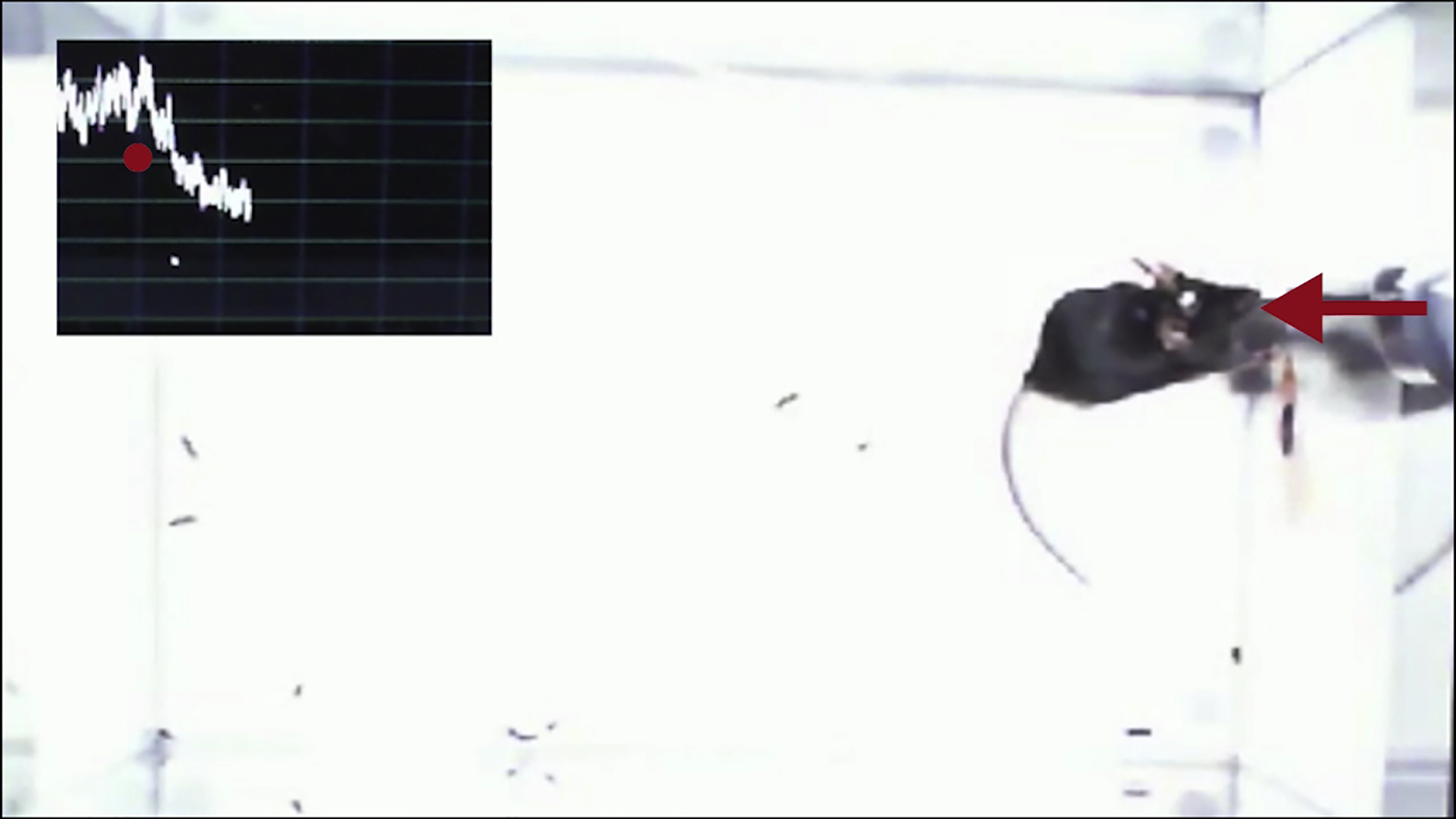

Supplement: Movie S1. Real-Time Recording of Orexin/Hypocretin Population Activity, Top Left, during a Continuous Licking Bout in a Freely Moving Mouse, Related to Figure 1 — The arrow shows the location of the food spout containing liquid food (strawberry milkshake). The red dot marks the time when the mouse starts licking. [file mmc2.jpg]
