# Supplementary material for: Inhibitory Interplay between Orexin Neurons and Eating
Source: Curr Biol. 2016 Sep 26;26(18):2486–91. doi: 10.1016/j.cub.2016.07.013 (PMC5049542; doi:10.1016/j.cub.2016.07.013)
Supplement: Document S2. Article plus Supplemental Information [file mmc3.pdf]

Current Biology

Report

Inhibitory Interplay between Orexin Neurons and Eating

Highlights

- Brain orexin cell activity is rapidly inactivated upon the act of eating
- This occurs with different foods, including calorie-free and liquid food
- Complete orexin cell inactivation in adult brain can promote overeating
- Overweight resulting from orexin cell loss is reversible by mild dieting

Authors

J. Antonio González, Lise T. Jensen, Panagiota Iordanidou, Molly Strom, Lars Fugger, Denis Burdakov

Correspondence

lars.fugger@imm.ox.ac.uk (L.F.),
denis.burdakov@kcl.ac.uk (D.B.)

In Brief

Brain orexin cells control arousal, but pathological deregulation of their activity can produce sleepiness, obesity, and anxiety. González et al. show that the voluntary act of eating can rapidly downregulate orexin cell activity irrespective of taste or calories, whereas obesity resulting from orexin underactivity can be reversed by a mild diet.

González et al., 2016, Current Biology *26*, 2486–2491
September 26, 2016 © 2016 The Author(s). Published by Elsevier Ltd.
http://dx.doi.org/10.1016/j.cub.2016.07.013

CellPress
Current Biology
Report

Inhibitory Interplay between Orexin Neurons and Eating

J. Antonio González,[1,6] Lise T. Jensen,[2,6] Panagiota Iordanidou,[1] Molly Strom,[3] Lars Fugger,[2,4,*] and Denis Burdakov[1,5,7,*]
[1]Mill Hill Laboratory, The Francis Crick Institute, London NW7 1AA, UK
[2]Institute of Clinical Medicine, Aarhus University, 8200 Aarhus, Denmark
[3]Sainsbury Wellcome Centre, University College London, London W1T 4JG, UK
[4]Oxford Centre for Neuroinflammation, Division of Clinical Neurology and MRC Human Immunology Unit, Nuffield Department of Clinical Neurosciences, Weatherall Institute of Molecular Medicine, University of Oxford, Oxford OX3 9DS, UK
[5]Institute of Psychiatry, Psychology and Neuroscience, Department of Developmental Neurobiology, King's College London, London WC2R 2LS, UK
[6]Co-first author
[7]Lead Contact
*Correspondence: lars.fugger@imm.ox.ac.uk (L.F.), denis.burdakov@kcl.ac.uk (D.B.)
http://dx.doi.org/10.1016/j.cub.2016.07.013

SUMMARY

In humans and rodents, loss of brain orexin/hypocretin (OH) neurons causes pathological sleepiness [1–4], whereas OH hyperactivity is associated with stress and anxiety [5–10]. OH cell control is thus of considerable interest. OH cells are activated by fasting [11, 12] and proposed to stimulate eating [13]. However, OH cells are also activated by diverse feeding-unrelated stressors [14–17] and stimulate locomotion and "fight-or-flight" responses [18–20]. Such OH-mediated behaviors presumably preclude concurrent eating, and loss of OH cells produces obesity, suggesting that OH cells facilitate net energy expenditure rather than energy intake [2, 21–23]. The relationship between OH cells and eating, therefore, remains unclear. Here we investigated this issue at the level of natural physiological activity of OH cells. First, we monitored eating-associated dynamics of OH cells using fiber photometry in free-feeding mice. OH cell activity decreased within milliseconds after eating onset, and remained in a down state during eating. This OH inactivation occurred with foods of diverse tastes and textures, as well as with calorie-free "food," in both fed and fasted mice, suggesting that it is driven by the act of eating itself. Second, we probed the implications of natural OH cell signals for eating and weight in a new conditional OH cell-knockout model. Complete OH cell inactivation in adult brain induced a hitherto unrecognized overeating phenotype and caused overweight that was preventable by mild dieting. These results support an inhibitory interplay between OH signals and eating, and demonstrate that OH cell activity is rapidly controllable, across nutritional states, by voluntary action.

RESULTS AND DISCUSSION

Natural Population Dynamics of OH Cells during Voluntary Eating

Orexin/hypocretin (OH) cells are activated by fasting and low glucose levels, and have been hypothesized to drive eating until ingested glucose slowly (within minutes) inactivates them (Figure 1A) [19, 24]. We measured OH cell population activity in freely behaving mice using fiber photometry [17] of the GCaMP6s activity indicator targeted to OH cells while monitoring eating using video tracking or touch sensors (Figure 1B; Figures S1–S3). In freely behaving mice, we observed activity fluctuations in OH-GCaMP6s, but not in OH-eGFP, cells (Figure 1C). The magnitude of these fluctuations (~10%–40% ΔF/F) was similar to network dynamics recorded with similar methods in other brain regions [25, 26]. Our experimental quantification of photometry performance suggested that >95% of the fluorescence signal would come from ~0.5 mm from the fiber tip (Figures S2A and S2B), which is well suited to OH cluster dimensions in the mouse hypothalamus. We confirmed that the GCaMP6s signal reflects physiological OH cell modulation by reproducing the previously described in vivo activation of OH cells by sounds [14] and in vitro inhibition of OH cells by glucose [24] (Figures S1C and S1D). The OH-GCaMP6s signal was directly proportional to the OH cell firing rate (Figure S3).

We found that food contact depressed OH cell activity extremely rapidly (<1 s of food contact) (Figures 1D–1H). OH cells returned to an up state within seconds after food contact was stopped (Figures 1D–1F; Figure S2E), suggesting that the rapid OH cell modulation is not caused by slowly varying nutritional signals. This effect was seen in both fasted and fed OH-GCaMP6s mice, but not in the OH-eGFP controls (Figure 1H). For liquid foods, the fall in OH cell activity was apparent within just a few licks (Figure 1F; Figures S2E and S2F). The eating-associated OH cell depression was similar for foods of differing consistency (e.g., chow versus yogurt) and different appetitive value (e.g., chow versus peanut butter) (Figure 1H). To confirm whether caloric content had a role, we tested a zero-calorie "food" (sucralose solution), and still observed robust OH cell inactivation during licking (Figure 1H). Overall, these data show

 © 2016 The Author(s). Published by Elsevier Ltd.
This is an open access article under the CC BY license (http://creativecommons.org/licenses/by/4.0/).

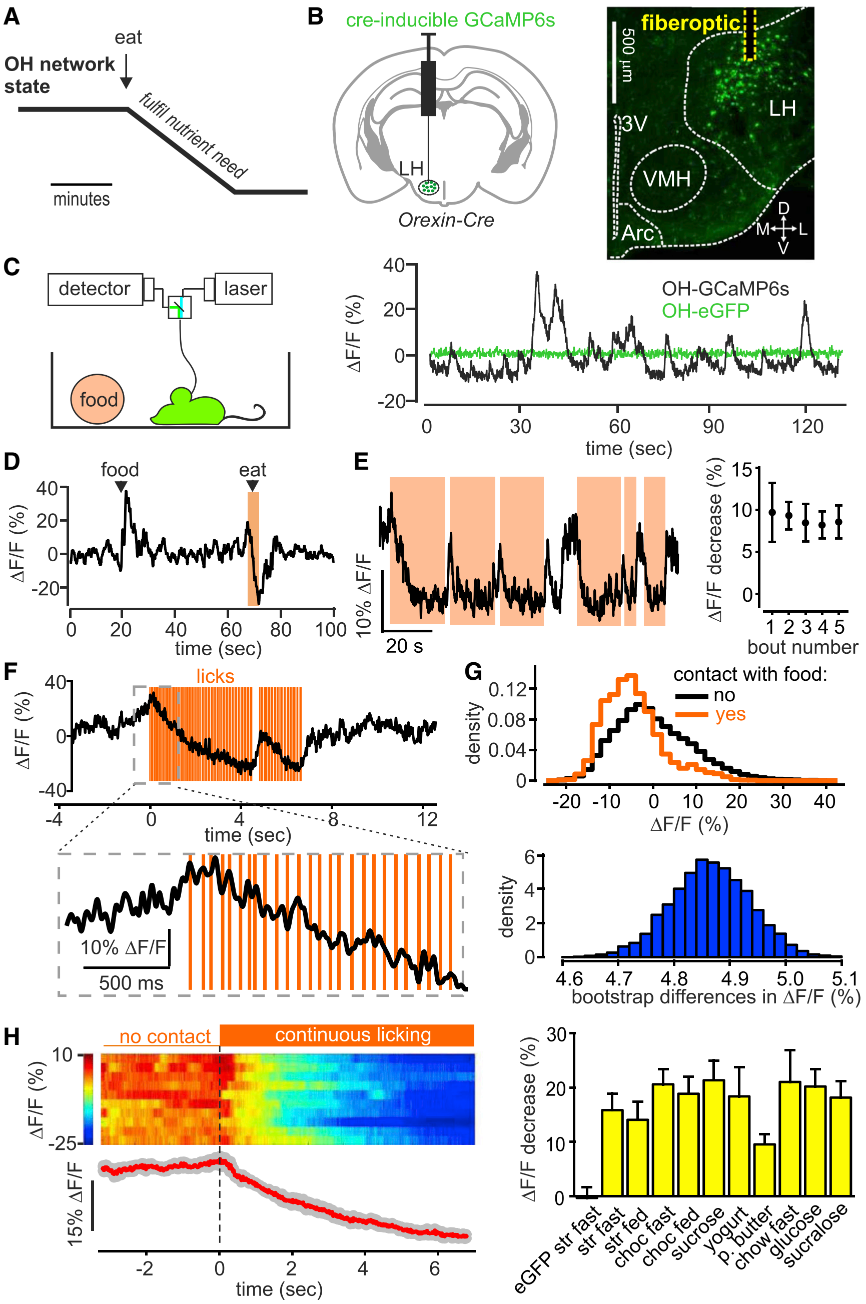

Figure 1. Impact of Eating on Natural OH Cell Dynamics In Vivo

(A) A hypothesis for temporal modulation of OH cells during eating.

(B) Left: targeting scheme of GCaMP6s to OH cells for obtaining the data shown in this figure (data using alternative targeting of OH cells are shown in Figures S2C–S2F). Right: localization of injection site and path of the optical fiber. 3V, third ventricle; L, D, M, V, lateral, dorsal, medial, ventral; VMH, ventromedial hypothalamus; Arc, arcuate nucleus. Representative image of n = 5 brains.

(C) Left: recording scheme. Right: fluorescence trace during cage exploration for mice expressing GCaMP6s or eGFP in OH neurons. Typical examples of n = 5 and n = 3 mice, respectively.

(D) Fluorescence trace during introduction of food into the cage and its subsequent consumption (orange-shaded area). Food was a drop of strawberry milkshake. Typical example of n = 5 mice.

(E) Left: fluorescence trace during repeated bouts of food contact (orange-shaded areas; food is strawberry milkshake). Typical example of n = 5 mice. Right: quantification of fluorescence change during the first 2 s of consecutive food-contact bouts (means ± SEM, n = 3 mice).

(F) Fluorescence change during food licking detected with a touch sensor (food is strawberry milkshake). Typical example of n = 5 mice across eight foods shown in (H), right.

(G) Top: probability density of OH cell activity. Bottom: distribution of the bootstrap differences of the same data. Typical example of n = 3 mice.

(H) Left: peri-event plots aligned to the onset of licking bouts (dashed line). The heatmap shows individual bouts (two per mouse), and the trace below the heatmap shows the mean of trial averages from each mouse (red line; gray lines represent SEM); n = 5 mice. Right: quantification of the experiment shown on the left, for different foods. Each column shows fluorescence change during the first 4 s of a licking bout (mean signals during 3.5 to 4 s minus signal during −0.5 to 0 s, times relative to the first lick). Data are means ± SEM of n = 4 mice in each group. Left column is control (OH-eGFP mice); other columns are OH-GCaMP6s mice; for food abbreviations, see the Supplemental Experimental Procedures; fast, overnight fasted before the experiment; fed, ad libitum feeding before the experiment. All changes in OH-GCaMP6s mice were significant ($p < 0.05$ in one-sample t tests of response to each food, DF = 3, $t > 3.4$).

See also Figures S1–S3 and Movie S1.

that OH cells are rapidly inactivated by the act of eating, irrespective of food properties or body energy state.

Natural Impact of OH Neurons on Eating

The above correlative data have two possible causal interpretations: (1) OH cells oppose eating, and are disabled to enable eating, or (2) OH cells drive eating, and so eating stops shortly after OH cells are silenced. To distinguish between these possibilities, we investigated causality between natural OH activity and eating by inactivating OH cells in adult mice through a toxin receptor-mediated cell-knockout strategy [27, 28].

We generated new transgenic mice in which the expression of the human diphtheria toxin receptor (DTR) is driven by the OH promoter (see the Supplemental Experimental Procedures). In OH-DTR mice, but not in control WT mice, the injection of diphtheria toxin ablated all OH cells, but not the neighboring melanin-concentrating hormone-containing cells, within a couple of days (Figures 2A–2D). This complete inactivation of OH cells, which is not as readily achievable through alternative silencing methods such as opto- and chemo-genetics, may be critical for elucidation of their full impact, because key deficiency phenotypes are not apparent upon partial inactivation [13].

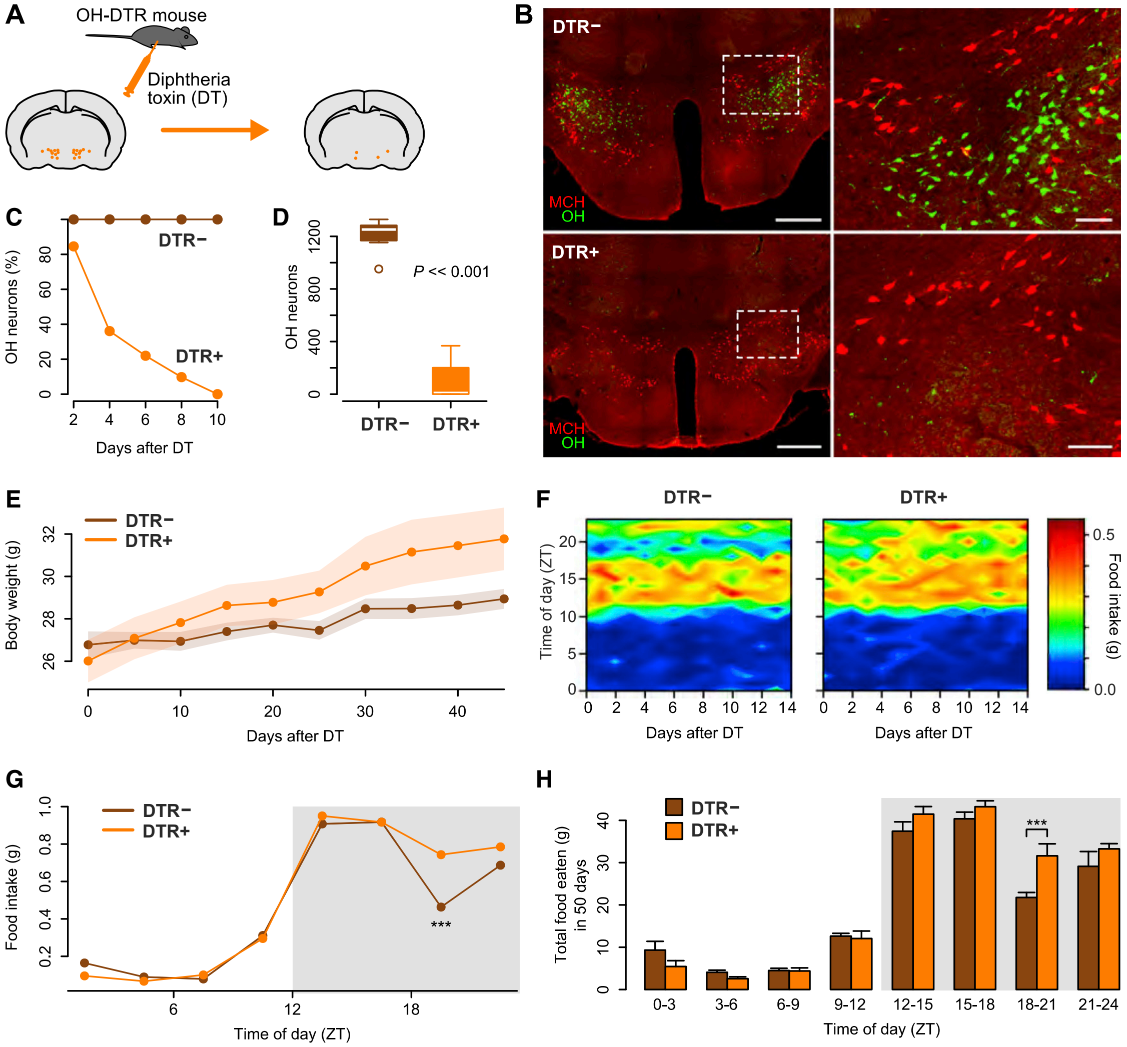

Figure 2. Impact of Natural OH Cell Activity on Spontaneous Feeding Rhythm

(A) Strategy for destroying OH neurons in adult mice.

(B) Immunostaining for OH (green) and melanin-concentrating hormone-containing (MCH) (red) neurons in DTR⁻ (top) and DTR⁺ (bottom) littermates 10 days after DT injection. The DT injection led to the loss of OH neurons in all brains tested (n = 8). Scale bars represent 500 µm (left) and 100 µm (right). Dashed boxes in the left-hand panels indicate the areas shown in corresponding right-hand panels.

(C) Time course of OH cell loss after DT injection in DTR^+ and their DTR^- littermates (n = 5 mice in each group).

(D) Quantification of OH cell number >21 days after DT injection in DTR^- and DTR^+ littermates. Unpaired t test, $t(13.3) = 16.41$, $p = 3.2e-10$, n = 8 mice in each group.

(E) Body weight time series of DTR^- and DTR^+ littermates after DT injection. ANCOVA, $F(1, 12) = 12.07$, $p = 0.005$, n = 7 mice in each group.

(F) Daily rhythm of eating in DTR^- and DTR^+ mice after DT injection, across days. n = 7 mice in each group.

(G) Mean daily rhythm of eating (average of 14 days; gray box is lights off) in DT-injected DTR^- and DTR^+ mice. Repeated-measures ANOVA, interaction: $F(7, 84) = 2.38$, $p = 0.029$. Significant differences were found only at the time of day indicated (***$p < 0.001$, Holm correction for multiple comparisons). n = 7 mice in each group.

(H) Total food consumed after DT injection, relative to the time of day. Repeated-measures ANOVA, interaction: $F(7, 84) = 3.07$, $p = 0.006$. Pairwise comparisons revealed statistical differences at the time of day indicated (***$p < 0.001$, Holm correction for multiple comparisons). n = 7 animals in each group.

See also Figure S4.

DT injection led to greater weight gain in DTR^+ mice than in their DTR^- littermates (Figure 2E), confirming that OH cells oppose overweight. Next, we probed food intake patterns at an hourly temporal resolution, using a food hopper specifically designed to re-capture any food spillage and a food-weighing system whose errors were sufficiently low to report changes greater than 0.01 g (Figures S4A–S4D). In DTR^- mice injected with DT (control mice) this revealed a robust daily eating rhythm, where food intake was largely restricted to the lights-off phase (“night”) but had a pronounced “dip” late at night (Figures 2F and 2G). However, in DTR^+ mice injected with DT, this dip in eating was significantly reduced, causing them to consume significantly more food during the late night (Figures 2F–2H; note that the magnitude of this overeating is well

CellPress

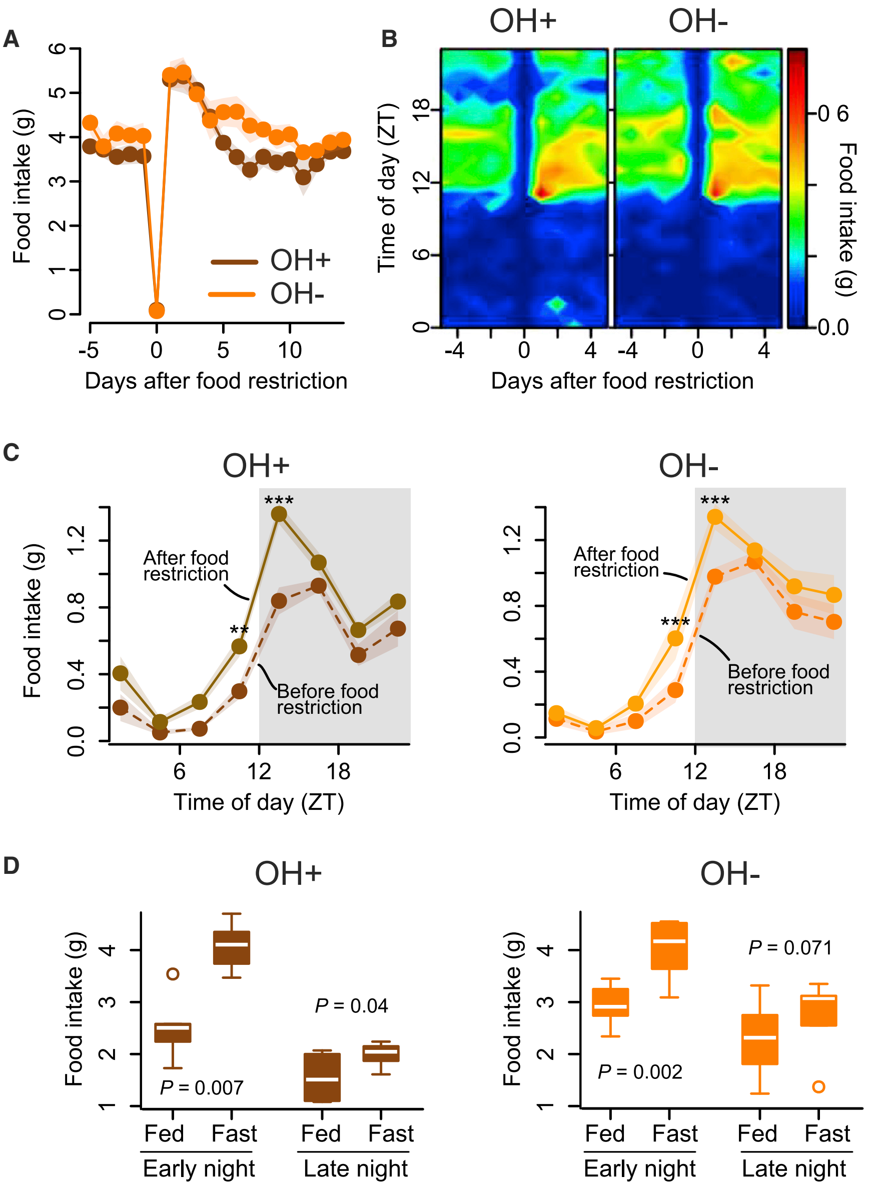

Figure 3. Impact of Natural OH Cell Activity on Rebound Eating after Fasting

(A) Eating responses to a 1-day fast in control mice (OH^+, DTR^- mice injected with DT) and their OH cell-deficient littermates (OH^-, DTR^+ mice injected with DT). n = 6 mice in each group.

(B) Daily eating rhythms before and after a 1-day fast in OH^+ and OH^- littermates. n = 6 mice in each group.

(C) Daily eating rhythms 3 days before and after food restriction. Repeated-measures ANOVA, interaction: $F(7, 35) = 3.36$, $p = 0.008$ (left) and $F(7, 35) = 3.53$, $p = 0.006$ (right). Follow-up tests showed significant differences only at times marked with asterisks (**$p < 0.01$, ***$p < 0.001$, Holm correction for multiple comparisons). n = 6 mice in each group.

(D) Total food consumed 3 days before (fed) and 3 days after (fast) food restriction in OH^+ and OH^- mice (n = 6 in each group) during early night (ZT12–14) compared to that consumed during late night (ZT18–20). Paired t tests.

above the sensitivity limit of the food-weight detector; Figures S4A–S4D). Interestingly, this overeating did not cause compensatory undereating at other times of day (Figures 2F–2H). Glucose tolerance in OH cell-deficient mice was normal (Figure S4E), as previously observed for OH-deficient humans [29], suggesting that OH cell loss does not prevent glucose uptake from blood into tissues. Overall, these data show that the natural OH cell activity prevents overeating and suppresses weight gain.

Temporal Dissociation of Fasting-Dependent and OH-Dependent Eating

The above findings demonstrate that the natural activity of OH cells opposes eating, and therefore challenge current models postulating that OH cells stimulate eating. However, because OH cells are activated by fasting, it is still possible that they become critical for compensatory eating after fasting [11]. To examine this possibility, we measured rebound overeating after fasting in OH^- mice (i.e., DTR^+ mice injected with DT), but surprisingly found it similar to their OH^+ littermates (DTR^- mice injected with DT) (Figures 3A and 3B). Furthermore, although both the OH^- and OH^+ mice overate for several days after fasting, on each of these days they ate more only in the early-night phase (Figures 3C and 3D), i.e., a different phase from that when food intake was naturally regulated by OH cells (Figure 2G). This temporal dissociation reveals that, at least under the conditions studied here, nutrient shortage and OH cells regulate distinct daily phases of food intake. These data suggest that OH cells are not required for compensatory overeating after fasting, at least when food is readily available.

Normalizing Eating Prevents Weight Gain in OH Cell-Deficient Mice

Identifying an effective strategy for body weight control in OH-deficient individuals is of clinical interest [21, 30]. In our experiments, the overeating in the OH^- mice (~10 g of food over 50 days or ~2% daily) could be theoretically sufficient to account for the overweight in these mice based on the following reasoning. The OH^- mice gain ~10% weight (relative to OH^+ controls) over about 45 days, i.e., ~0.2% daily excess weight gain (Figure 2E). Our fasting experiment shows that, in 1 day, a 100% change in food intake can cause a 10% change in weight (Figure 3A). If this 10:1 relation holds in general, then the ~2% daily eating increase would be sufficient to account for the 0.2% daily excess weight gain.

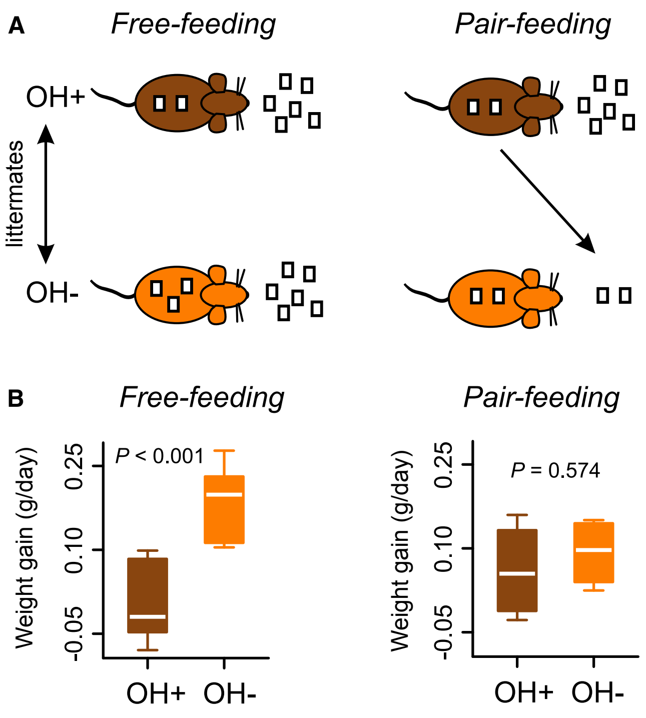

Figure 4. Prevention of Weight Gain Caused by OH Cell Loss by Dieting

(A) Strategy for pair-feeding experiment (OH^+, DTR^- mice injected with DT; OH^-, their DTR^+ littermates injected with DT).

(B) Weight gain of OH^- and OH^+ littermates during weeks 2 and 3 after DT injection, and during free feeding (unpaired t test, $t(11.93) = -4.327$, $p = 0.0009$, $n = 7$ mice in each group) and pair feeding (unpaired t test, $t(5.41) = -0.598$, $p = 0.574$, $n = 4$ animals in each group).

Therefore, we investigated whether the excess weight gain in the OH^- mice can be controlled by mild dietary restriction. To achieve this, we employed the pair-feeding paradigm [31], where the food available to each OH^- mouse was matched daily to that eaten by its OH^+ littermate control (Figure 4A). This effectively fixes food intake, but in a mild physiological manner without abnormal periods of fasting [31].

Pair feeding the OH^- mice prevented them from becoming heavier than their OH^+ siblings (Figure 4B). In contrast, in control experiments performed during the same time, free-feeding OH^- mice became significantly heavier than their OH^+ siblings (Figure 4B). Note that this does not exclude that factors other than eating may mediate weight gain of OH^- mice, for example, alterations in sleep/wake rhythms and energy expenditure [2, 31, 32]. However, our results do suggest that, in the absence of overeating, such factors are insufficient to cause the excessive weight gain.

Conclusions

Our findings reveal population activity dynamics of OH cells during eating, and suggest that eating is less likely to occur when OH cells are naturally active. OH cells stimulate "non-eating" behaviors such as locomotion [18]. Although these behaviors expend energy, it is difficult to eat while performing them. Thus, a possible evolutionary rationale for our findings is that suppression of OH-dependent non-eating behaviors—by silencing OH cells upon food contact—would facilitate eating (e.g., by making the mouse less likely to move away from the food). It is tempting to speculate that increased eating after experimental stimulation of the OH system [11, 13] may be a secondary response to OH-driven energy expenditure [33], rather than a primary function of OH cells.

Our findings identify interesting directions for future work. We found that the weight gain in OH-cell-deficient mice could be prevented by mild caloric restriction. This suggests that development of non-pharmacological interventions may be useful in managing excessive weight gain in neuropsychiatric conditions associated with reduced OH signaling [3, 21, 30, 34]. Furthermore, our results suggest that OH cells—whose hyperactivity has been implicated in pathological states such as panic anxiety [5]—could be inhibited by simple voluntary actions such as eating, irrespective of the nature of the food. Further research into neuroscience-based lifestyle interventions for anxiety and obesity might lead to treatments that are easier to implement and have fewer side effects.

EXPERIMENTAL PROCEDURES

OH cell activity was recorded in vivo using fiber photometry of the GCaMP6s activity indicator targeted to OH cells either using previously characterized orexin-Cre mice and Cre-inducible GCaMP6s viral vectors [35, 36] (Figure 1; Figure S1B) or using a newly generated orexin promoter-dependent adeno-associated virus (AAV)-GCaMP6s vector (characterized in Figures S2C–S2F and described in the Supplemental Experimental Procedures). OH cells were specifically and completely inactivated using a diphtheria toxin receptor-mediated cell-ablation strategy in newly generated OH-DTR transgenic mice described in the Supplemental Experimental Procedures. Food intake was monitored using a TSE PhenoMaster system, whose sensitivity and accuracy were directly determined in our laboratory (Figures S4A–S4D). Immunohistochemistry and glucose tolerance tests were performed using standard techniques (see the Supplemental Experimental Procedures).

SUPPLEMENTAL INFORMATION

Supplemental Information includes Supplemental Experimental Procedures, four figures, and one movie and can be found with this article online at http://dx.doi.org/10.1016/j.cub.2016.07.013.

AUTHOR CONTRIBUTIONS

J.A.G. conducted most of the experiments; L.T.J. created and characterized the DTR transgenic mice; P.I. conducted the experiments in Figures S2C–S2F; M.S. contributed to the experiments in Figure S2C; D.B. and L.F. designed the study and obtained funding; and D.B., L.F., and L.T.J. wrote the paper.

ACKNOWLEDGMENTS

We thank Drs. Cornelia Schöne, Rui Costa, and Guohong Cui for their assistance with fiber photometry. This work was funded by The Francis Crick Institute, which receives its core funding from Cancer Research UK, the UK Medical Research Council, and the Wellcome Trust. L.F. is supported by the MRC and the Wellcome Trust.

Received: June 15, 2016
Revised: July 5, 2016
Accepted: July 8, 2016
Published: August 18, 2016

REFERENCES

1. Chemelli, R.M., Willie, J.T., Sinton, C.M., Elmquist, J.K., Scammell, T., Lee, C., Richardson, J.A., Williams, S.C., Xiong, Y., Kisanuki, Y., et al. (1999). Narcolepsy in orexin knockout mice: molecular genetics of sleep regulation. Cell *98*, 437–451.
2. Hara, J., Beuckmann, C.T., Nambu, T., Willie, J.T., Chemelli, R.M., Sinton, C.M., Sugiyama, F., Yagami, K., Goto, K., Yanagisawa, M., and Sakurai, T. (2001). Genetic ablation of orexin neurons in mice results in narcolepsy, hypophagia, and obesity. Neuron *30*, 345–354.
3. Thannickal, T.C., Moore, R.Y., Nienhuis, R., Ramanathan, L., Gulyani, S., Aldrich, M., Cornford, M., and Siegel, J.M. (2000). Reduced number of hypocretin neurons in human narcolepsy. Neuron *27*, 469–474.
4. Nishino, S., Ripley, B., Overeem, S., Lammers, G.J., and Mignot, E. (2000). Hypocretin (orexin) deficiency in human narcolepsy. Lancet *355*, 39–40.
5. Johnson, P.L., Truitt, W., Fitz, S.D., Minick, P.E., Dietrich, A., Sanghani, S., Träskman-Bendz, L., Goddard, A.W., Brundin, L., and Shekhar, A. (2010). A key role for orexin in panic anxiety. Nat. Med. *16*, 111–115.
6. Suzuki, M., Beuckmann, C.T., Shikata, K., Ogura, H., and Sawai, T. (2005). Orexin-A (hypocretin-1) is possibly involved in generation of anxiety-like behavior. Brain Res. *1044*, 116–121.
7. Li, Y., Li, S., Wei, C., Wang, H., Sui, N., and Kirouac, G.J. (2010). Orexins in the paraventricular nucleus of the thalamus mediate anxiety-like responses in rats. Psychopharmacology (Berl.) *212*, 251–265.
8. Johnson, P.L., Molosh, A., Fitz, S.D., Truitt, W.A., and Shekhar, A. (2012). Orexin, stress, and anxiety/panic states. Prog. Brain Res. *198*, 133–161.
9. Heydendael, W., Sengupta, A., Beck, S., and Bhatnagar, S. (2014). Optogenetic examination identifies a context-specific role for orexins/hypocretins in anxiety-related behavior. Physiol. Behav. *130*, 182–190.
10. Boutrel, B., Kenny, P.J., Specio, S.E., Martin-Fardon, R., Markou, A., Koob, G.F., and de Lecea, L. (2005). Role for hypocretin in mediating stress-induced reinstatement of cocaine-seeking behavior. Proc. Natl. Acad. Sci. USA *102*, 19168–19173.
11. Sakurai, T., Amemiya, A., Ishii, M., Matsuzaki, I., Chemelli, R.M., Tanaka, H., Williams, S.C., Richardson, J.A., Kozlowski, G.P., Wilson, S., et al. (1998). Orexins and orexin receptors: a family of hypothalamic neuropeptides and G protein-coupled receptors that regulate feeding behavior. Cell *92*, 573–585.
12. Diano, S., Horvath, B., Urbanski, H.F., Sotonyi, P., and Horvath, T.L. (2003). Fasting activates the nonhuman primate hypocretin (orexin) system and its postsynaptic targets. Endocrinology *144*, 3774–3778.
13. Inutsuka, A., Inui, A., Tabuchi, S., Tsunematsu, T., Lazarus, M., and Yamanaka, A. (2014). Concurrent and robust regulation of feeding behaviors and metabolism by orexin neurons. Neuropharmacology *85*, 451–460.
14. Mileykovskiy, B.Y., Kiyashchenko, L.I., and Siegel, J.M. (2005). Behavioral correlates of activity in identified hypocretin/orexin neurons. Neuron *46*, 787–798.
15. Williams, R.H., Jensen, L.T., Verkhratsky, A., Fugger, L., and Burdakov, D. (2007). Control of hypothalamic orexin neurons by acid and CO2. Proc. Natl. Acad. Sci. USA *104*, 10685–10690.
16. Sunanaga, J., Deng, B.S., Zhang, W., Kanmura, Y., and Kuwaki, T. (2009). CO2 activates orexin-containing neurons in mice. Respir. Physiol. Neurobiol. *166*, 184–186.
17. González, J.A., Iordanidou, P., Strom, M., Adamantidis, A., and Burdakov, D. (2016). Awake dynamics and brain-wide direct inputs of hypothalamic MCH and orexin networks. Nat. Commun. *7*, 11395.
18. Hagan, J.J., Leslie, R.A., Patel, S., Evans, M.L., Wattam, T.A., Holmes, S., Benham, C.D., Taylor, S.G., Routledge, C., Hemmati, P., et al. (1999). Orexin A activates locus coeruleus cell firing and increases arousal in the rat. Proc. Natl. Acad. Sci. USA *96*, 10911–10916.
19. Yamanaka, A., Beuckmann, C.T., Willie, J.T., Hara, J., Tsujino, N., Mieda, M., Tominaga, M., Yagami, K., Sugiyama, F., Goto, K., et al. (2003). Hypothalamic orexin neurons regulate arousal according to energy balance in mice. Neuron *38*, 701–713.
20. Kuwaki, T., and Zhang, W. (2012). Orexin neurons and emotional stress. Vitam. Horm. *89*, 135–158.
21. Nishino, S., Ripley, B., Overeem, S., Nevsimalova, S., Lammers, G.J., Vankova, J., Okun, M., Rogers, W., Brooks, S., and Mignot, E. (2001). Low cerebrospinal fluid hypocretin (Orexin) and altered energy homeostasis in human narcolepsy. Ann. Neurol. *50*, 381–388.
22. Tabuchi, S., Tsunematsu, T., Black, S.W., Tominaga, M., Maruyama, M., Takagi, K., Minokoshi, Y., Sakurai, T., Kilduff, T.S., and Yamanaka, A. (2014). Conditional ablation of orexin/hypocretin neurons: a new mouse model for the study of narcolepsy and orexin system function. J. Neurosci. *34*, 6495–6509.
23. Nevsimalova, S., Vankova, J., Stepanova, I., Seemanova, E., Mignot, E., and Nishino, S. (2005). Hypocretin deficiency in Prader-Willi syndrome. Eur. J. Neurol. *12*, 70–72.
24. Burdakov, D., Gerasimenko, O., and Verkhratsky, A. (2005). Physiological changes in glucose differentially modulate the excitability of hypothalamic melanin-concentrating hormone and orexin neurons in situ. J. Neurosci. *25*, 2429–2433.
25. Cui, G., Jun, S.B., Jin, X., Pham, M.D., Vogel, S.S., Lovinger, D.M., and Costa, R.M. (2013). Concurrent activation of striatal direct and indirect pathways during action initiation. Nature *494*, 238–242.
26. Gunaydin, L.A., Grosenick, L., Finkelstein, J.C., Kauvar, I.V., Fenno, L.E., Adhikari, A., Lammel, S., Mirzabekov, J.J., Airan, R.D., Zalocusky, K.A., et al. (2014). Natural neural projection dynamics underlying social behavior. Cell *157*, 1535–1551.
27. Saito, M., Iwawaki, T., Taya, C., Yonekawa, H., Noda, M., Inui, Y., Mekada, E., Kimata, Y., Tsuru, A., and Kohno, K. (2001). Diphtheria toxin receptor-mediated conditional and targeted cell ablation in transgenic mice. Nat. Biotechnol. *19*, 746–750.
28. Luquet, S., Perez, F.A., Hnasko, T.S., and Palmiter, R.D. (2005). NPY/AgRP neurons are essential for feeding in adult mice but can be ablated in neonates. Science *310*, 683–685.
29. Beitinger, P.A., Fulda, S., Dalal, M.A., Wehrle, R., Keckeis, M., Wetter, T.C., Han, F., Pollmächer, T., and Schuld, A. (2012). Glucose tolerance in patients with narcolepsy. Sleep *35*, 231–236.
30. Fortuyn, H.A., Swinkels, S., Buitelaar, J., Renier, W.O., Furer, J.W., Rijnders, C.A., Hodiamont, P.P., and Overeem, S. (2008). High prevalence of eating disorders in narcolepsy with cataplexy: a case-control study. Sleep *31*, 335–341.
31. Ellacott, K.L., Morton, G.J., Woods, S.C., Tso, P., and Schwartz, M.W. (2010). Assessment of feeding behavior in laboratory mice. Cell Metab. *12*, 10–17.
32. Zhang, S., Zeitzer, J.M., Sakurai, T., Nishino, S., and Mignot, E. (2007). Sleep/wake fragmentation disrupts metabolism in a mouse model of narcolepsy. J. Physiol. *581*, 649–663.
33. Teske, J.A., and Mavanji, V. (2012). Energy expenditure: role of orexin. Vitam. Horm. *89*, 91–109.
34. Brundin, L., Björkqvist, M., Petersén, A., and Träskman-Bendz, L. (2007). Reduced orexin levels in the cerebrospinal fluid of suicidal patients with major depressive disorder. Eur. Neuropsychopharmacol. *17*, 573–579.
35. Matsuki, T., Nomiyama, M., Takahira, H., Hirashima, N., Kunita, S., Takahashi, S., Yagami, K., Kilduff, T.S., Bettler, B., Yanagisawa, M., and Sakurai, T. (2009). Selective loss of GABA(B) receptors in orexin-producing neurons results in disrupted sleep/wakefulness architecture. Proc. Natl. Acad. Sci. USA *106*, 4459–4464.
36. Schöne, C., Cao, Z.F., Apergis-Schoute, J., Adamantidis, A., Sakurai, T., and Burdakov, D. (2012). Optogenetic probing of fast glutamatergic transmission from hypocretin/orexin to histamine neurons in situ. J. Neurosci. *32*, 12437–12443.

Current Biology, Volume *26*

Supplemental Information

Inhibitory Interplay between Orexin Neurons and Eating

J. Antonio González, Lise T. Jensen, Panagiota Iordanidou, Molly Strom, Lars Fugger, and Denis Burdakov

Supplemental Figure 1

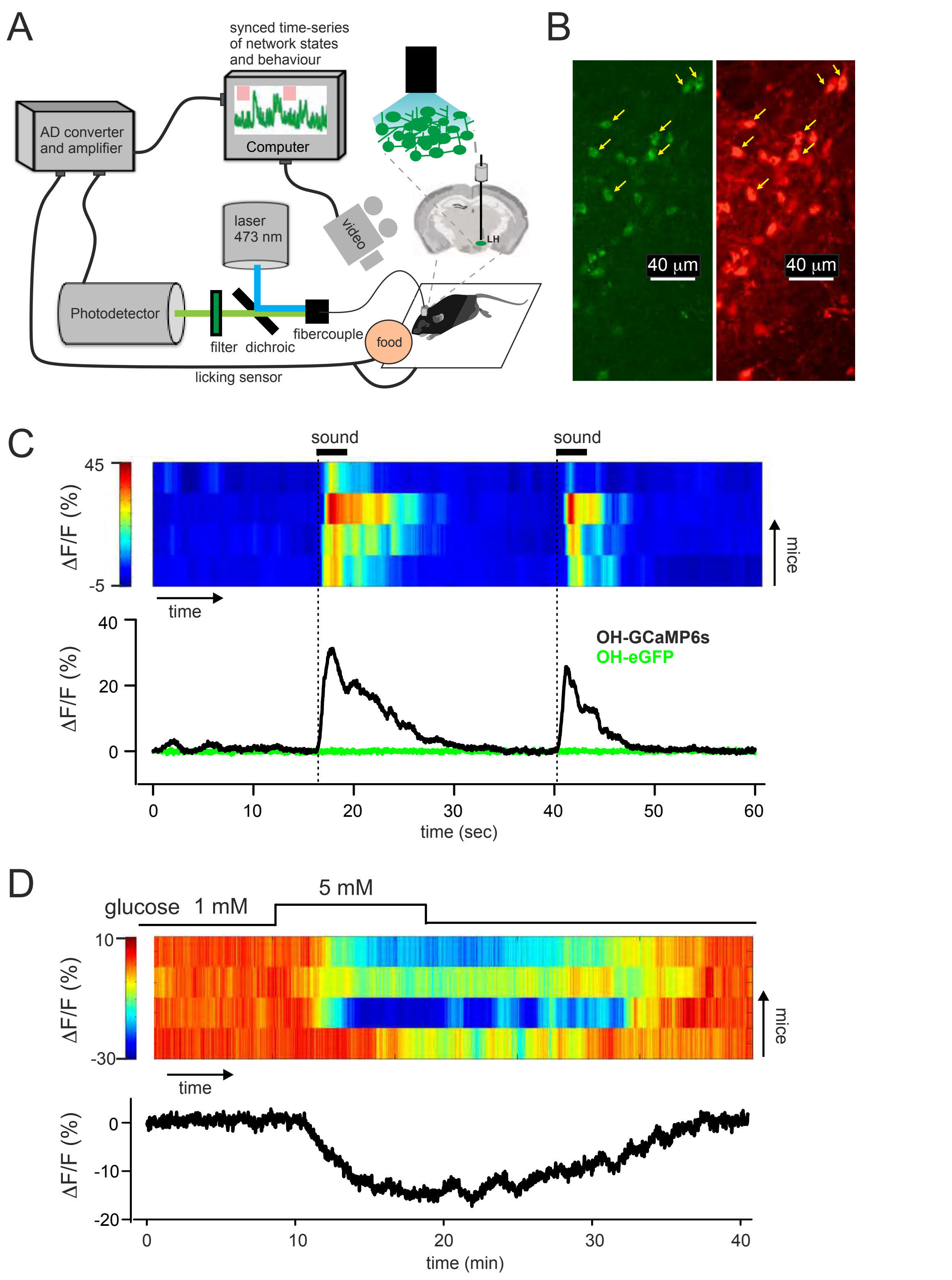

Supplemental Figure S1 (related to Figure 1)

A. Detailed scheme for co-registering neural activity and eating.

B. OH-GCaMP6s neurons (left, green) immunostained for OH (right, red) in an LH brain slice; some of the corresponding cells are arrowed.

C. Fiber photometry recording of fluorescence responses of freely-moving mice to a sudden novel sound (16 kHz, 100 db), in OH-GCaMP6s mice (n = 4, single trials from individual mice shown on heat-map, averaged responses shown below heat-map), and in OH-eGFP mice (n = 3 mice, black trace is averaged response).

D. Fiber photometry recordings of fluorescence responses in acutely isolated brain slices of OH-GCaMP6s cells to changes in ambient glucose concentration. Brain slices were prepared, and extracellular glucose level was manipulated, as in reference S3. The fiber tip was placed 100-300 µm above OH-GCaMP6s neurons in the lateral hypothalamus.
Heat-map shows individual trials, plot below the heat map shows average of the trials.

Supplemental Figure 2

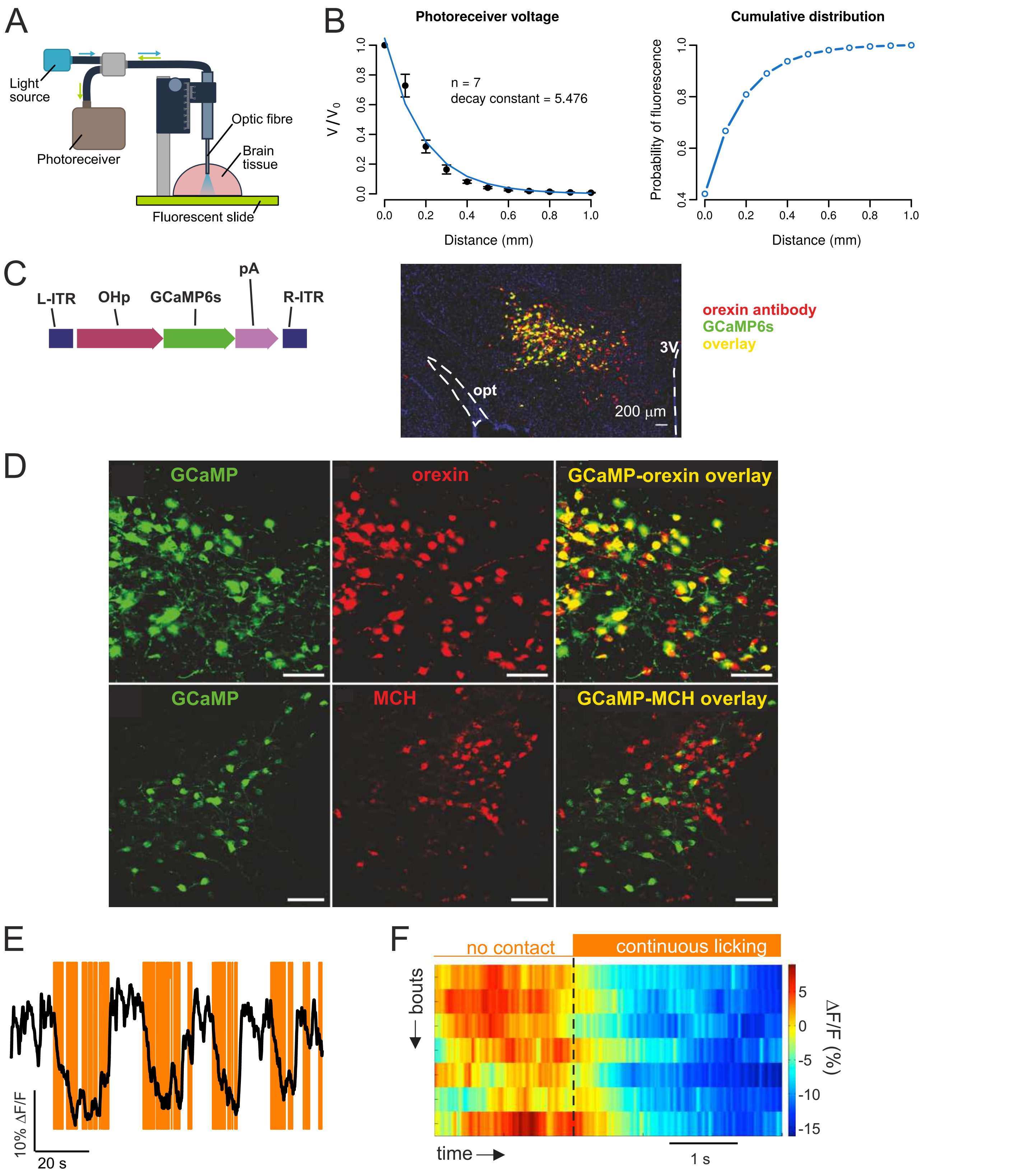

Supplemental Figure S2 (related to Figure 1). Characterization of fluorescence capture by the photometry fiber, and experiments using GCaMP6s expressed under control of the preproorexin promoter.

A. Scheme of experimental set-up. To characterize light capture from fluorescent objects in brain tissue, we measured fluorescence from a slide with an optic fiber placed at increasingly greater distances. The slide had similar fluorescence emission to GCaMP6.

B. *Left*, normalized fluorescence signal detected at different distances from the fiber tip. *Right*, Cumulative probability of fluorescence as a function of distance from the tip (calculated from data on the left). This shows that > 95% of fluorescence comes from within 0.5 mm depth from the fiber tip, which corresponds to a volume of 0.048 mm^3 (volume calculated as a conical frustum for a fiber of NA 0.37 and 0.2 mm diameter, and brain refractive index of 1.369 based on Sun et al., 2012, Opt Express 20: 1084-1095).

C. *Left,* Schematic of AAV construct. L- and R- ITR, left- and right- inverted terminal repeat; OHp, preproorexin promoter; pA, human growth hormone polyA. *Right*, Immunohistochemistry of the lateral hypothalamus (LH) of a WT C57BL6 mouse injected with the AAV2/1-OH-GCaMP6s virus. Orexin (Santa Cruz goat anti-orexin-A, 1:2000) labelling is in red and GCaMP6s in green. DAPI is shown in blue. Opt, optic tract; V3, third ventricle.

D. *Top row*, Same as C (right) at higher zoom: orexin immunoreactivity was detectable in 96.3% (1779/1848 cells from 3 brains) of GCaMP6s neurons. *Bottom row,* Melanin concentrating hormone (Phoenix pharmaceuticals, rabbit anti-MCH, 1:2000) labelling in a mouse injected with AAV2/1-OH-GCaMP6s virus. No co-localization of GCaMP6s and MCH immunoreactivity was observed. Scale bars, 100 µm.

E. Fiber photometry of OH-GCaMP6s fluorescence signal (black) during strawberry milkshake licking (orange). Experimental set-up is the same as in Fig. 1F, except GCaMP6s targeting was performed as in panels A-B above. Typical example of n = 4 mice.

F. Peri-event plots aligned to onset of licking bouts (dashed line). Heatmap shows consequitive licking bouts from the same mouse. Experimental set-up is the same as in E. Typical example of n = 4 mice.

Supplemental Figure 3

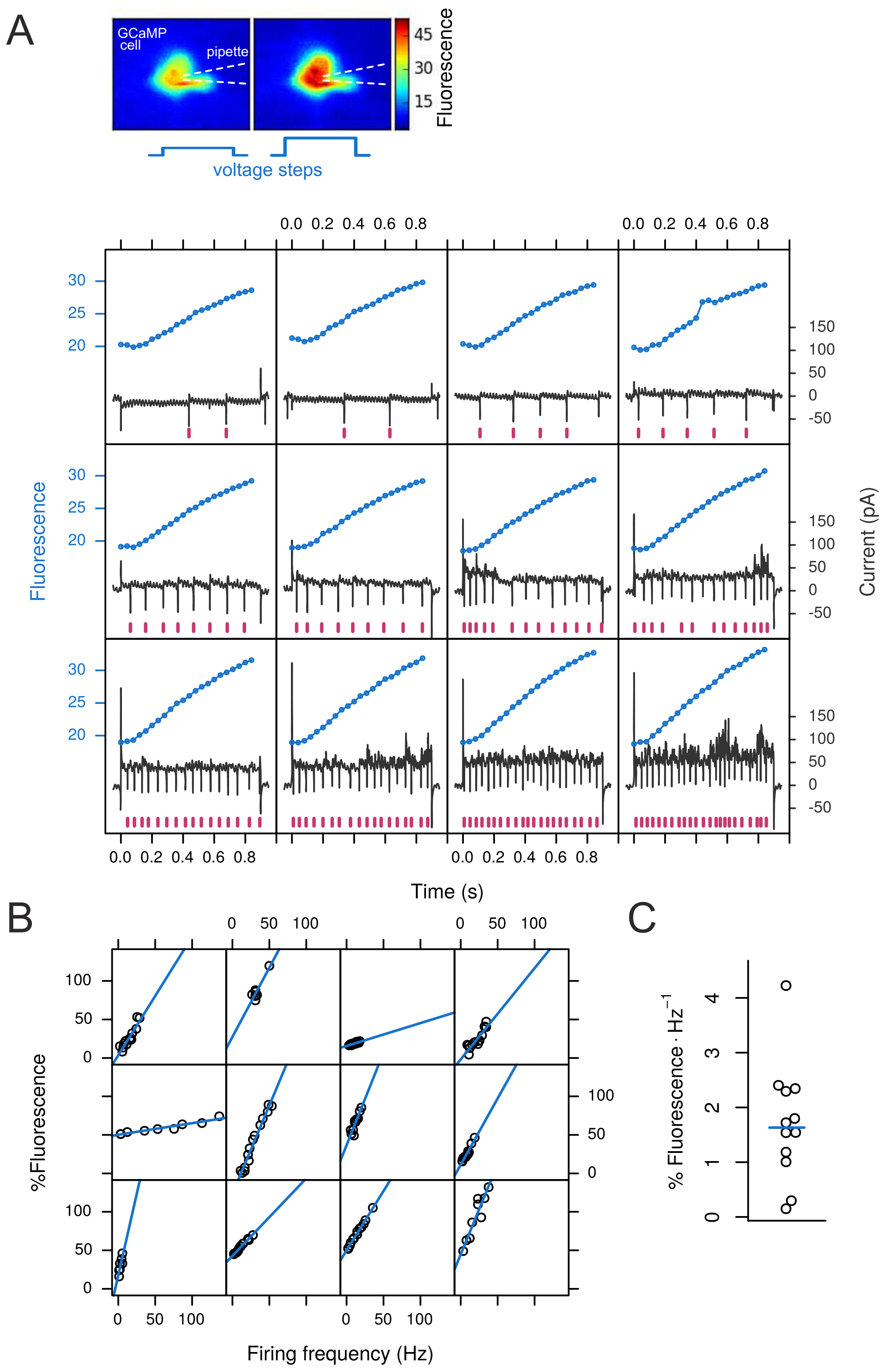

Supplemental Figure S3 (related to Figure 1).
GCaMP6s fluorescence changes associated with OH-GCaMP6s cell firing changes.

A. Top, raw images from the experiment. Bottom, examples of GCaMP6s recordings at different firing states (method described in Supplementary Methods, Section 1). Fluorescence is reported in raw pixel values (blue) and action potentials are detected (magenta) from the raw electrophysiology traces (black). Typical example of n = 12 cells.

B. Examples of the relationship between GCaMP6s signal and action potential firing in 12 different OH-GCaMP6s cells. Percent fluorescence was calculated from raw signals, such as those shown in A, as dF/F0 x 100, where F0 is the fluorescence at the beginning of the depolarizing pulse and dF is the difference in fluorescence between the end and the beginning of that same pulse.

C. Change in GCaMP6s fluorescence per Hz of firing rate for 12 OH-GCaMP6s cells, obtained from the slope of the linear fit of data in B. The median value (blue line) represents 1.6% fluorescence change for every 1 Hz firing change.

Supplemental Figure 4

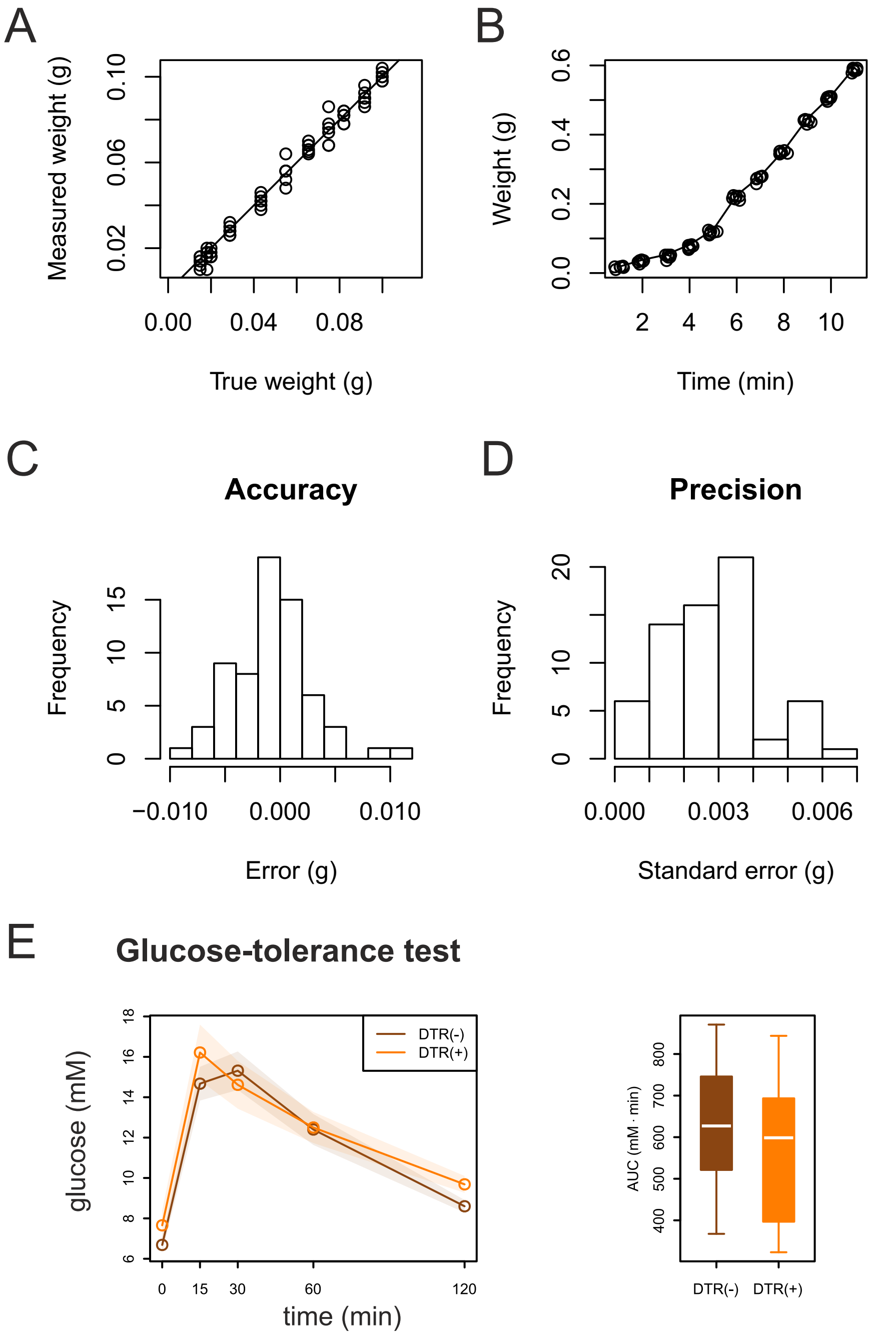

Supplemental Figure S4 (Related to Figure 2)
Determination of feeding sensor sensitivity. Each sensor was calibrated as usual and the food container was filled up. Then, a set of weights (n = 11, range 15 to 100 mg) were removed in sequence from the food container to simulate food intake. This test was repeated 5 times for each of 6 feeding sensors for a total of 330 measurements.

A. Relationship between true weight (line) and measured weight (points).
B. Temporal relationship between cumulative true weight (line) and measured weight (points), showing that weight can be accurately recorded on the time-scale of minutes.
C. Accuracy, illustrated as the frequency distribution of errors (differences between true and measured weight). The system is accurate to 0.01g.
D. Precision (a measure of repeatability), representing how close measurements made under the same conditions are to each other. The mean standard error is below 0.008 g.
E. Glucose tolerance analysis in mice with OH cells, DTR(-), and mice without OH cells, DTR(+); data are means and SEM of n = 7 mice (see Supplementary Methods).

SUPPLEMENTAL EXPERIMENTAL PROCEDURES

Targeting activity indicators to orexin/hypocretin neurons

Animal procedures were performed following UK Home Office regulations, and adult male mice were used in all experiments.

For calcium imaging, activity indicator GCaMP6s was targeted to OH neurons using two alternative methods. In Method 1 (Fig. 1, Fig. S1), AAVs carrying Cre-dependent GCaMP6s (rAAV9.CAG.Flex.GCaMP6s.WPRE.SV40; Penn Vector Core) or eGFP (AAV1.CAG.Flex.eGFP; UNC Vector Core) were stereotaxically injected into LH of orexin-Cre transgenic mice [S1, S2]. The specificity of Cre targeting to OH cells in this orexin-Cre line was quantified and described elsewhere [S1], and confirmed here (Fig. S1B). Cre-dependency of viral expression was confirmed by injections of the Cre-dependent viruses into the brains of Cre-negative C57BL6 mice (n = 3-6 mice for each virus).

Alternatively (Method 2, illustrated in Fig. S2E-F), GCaMP6s was targeted to OH neurons of WT C57BL6 mice using AAV vector that expresses GCaMP6s under the control of the preproorexin promoter (kind gift of Takeshi Sakurai). This promoter has been previously shown to target OH cells with high fidelity [S3], which is confirmed here (Fig. S2C-D). To make this AAV vector, we amplified the preproorexin promoter using oligonucleotides 5'–GTTCCTGCGGCCGCACGCGTGAGCTCAATAAAGAGGTT–3' and 5'–GGCAAGCTTCTGCAGGTCGACGGTGTCTGGCGC – 3', and cloned it into the MluI and SalI sites of pAAV-MCS (Agilent Technologies) using In-Fusion HD Cloning kit (Clontech), to create pAAV-OH. GCaMP6s gene was amplified from plasmid pGP-CMV-GCaMP6s (Addgene Plasmid #40753; [S4]) using oligonucleotides: 5'–TGAGCGCCAGACACCGTCGACCATGGGTTCTCATCAT–3' and 5'– GGGATGCCACCCGTAGATCTTCACTTCGCTGTCATCAT – 3' and cloned into pAAV-OH vector SalI and BglII sites as above to create pAAV-OH-GCaMP6s. Sequences of preproorexin promoter and GCamp6s were verified by sequencing using several internal oligonucleotides. AAV virus was prepared as described in [S5].

Three 50 nl injections of the Cre-dependent or the preproorexin promoter -dependent GCamp6s virus were made into orexin-Cre or WT C57BL6 mice respectively, at the following coordinates: 1.35 mm caudal from bregma; ±0.9 mm lateral from midline; and 5.30, 5.20, and 5.10 mm ventral from brain surface. A fiberoptic implant was stereotaxically installed with the fiber tip above the lateral hypothalamus, and fixed to the skull as previously described [S6].

In Fig. S3, to investigate the relationship between GCaMP6s signals and OH cell firing, we used cell-attached electrophysiology together with epifluorescence recording, in acute mouse brain slices. OH-Cre cells were tagged with GCaMP6s as described above, and their activity was measured using the cell-attached recording in voltage-clamp mode [S7]. Depolarizing voltage steps were applied to the cell, and GCaMP6s signals were elicited using a xenon excitation lamp and standard eGFP filters, and captured at 25 fps using a DAGE-MTI camera.

Fiber photometry

A fiber photometry setup was built according to general principles outlined in Cui et al., 2013, except a single multimode fiber was used for excitation and emission inside the brain as in [S8]. Specifically, the excitation light was sent into a fiber-coupled cube containing a dichroic mirror and GFP filters (Doric, FMC_GFP_FC), and from there into a patchchord (Doric, MFP_200/230/900-0.22_2m_FCM-MF) plugged into the fiberoptic implant (Doric, MFC_200/260-0.37_50mm_MF2.5(7.5mm)_FLT) using a brass connector (Doric, SLEEVE_BR_2.5). A 473 nm laser (Becker & Hickl) provided the excitation light. The laser power at the implant fiber tip was measured before implantation (X-Cite Optical Power Measurement System, Excelitas Technologies) and adjusted to 0.1 mW. Emitted fluorescence was fiber-coupled from the GFP cube to a photodetector (Becker & Hickl, HPM-CON-2). The analogue detector signal was sent to an AD port of a HEKA EPC-10 amplifier. The fluorescence signal was recorded using software provided with the amplifier (HEKA Patchmaster). Photometry data underwent minimal processing, consisting of standard within-trial normalization, in which the signal ΔF/F was defined as $100*(F-F_{mean}) / F_{mean} - 100$, where F is the raw fluorescence signal and F_{mean} is the mean fluorescence intensity of a 10 min baseline period before trial. Fiber tips were implanted at the following coordinates: 1.38 mm caudal from bregma; 0.95 mm lateral from midline; and 5 mm ventral from brain surface. The LH location of fiber tips were verified post-recording by examining slices with visible fiber tract; because most of these slices were damaged, intact slices closest to the fiber tip in the rostrocaudal plane were used for Fig. 1B, and the fiber tract was drawn.

Eating behavior quantification

Animals were kept on a 12h light-dark cycle (lights on at 7am). To control for circadian factors, the experiments were performed either during the dark phase (9pm-11pm) or the light phase (12pm-5pm); no differences in the described responses were observed between these circadian phases. Eating was defined as food-mouth touch, and its occurrence was precisely quantified by a custom touch sensor for wet food, built according to previously described specifications [S9]. The temporal relation between fluorescence and touch was preserved by collecting and processing the two signals simultaneously on the same AD board. Occasionally, the time of food contact was estimated from video-recordings time-synced to the photometry trace (Fig. 1E, Supplemental Movie 1). Foods examined in experiments shown in Fig. 1H were: str = strawberry milkshake (Yazoo), choc = chocolate milkshake (Yazoo), p.butter = peanut butter (Essential Waitrose), chow = wet crushed standard mouse chow, sucrose (0.4 M), sucralose (1.5 mM), yogurt (Yeo Valley plain), glucose (0.3 M). The examples shown in Fig. 1F, 1H (left) are from strawberry milkshake. Mice were habituated to all foods to remove effects of novelty.

Generation of orexin-DTR transgenic mice

As mice are insensitive to diphtheria toxin (DT), we generated transgenic mice expressing the human diphtheria toxin receptor driven (DTR) by the OH promoter in order to perform selective killing of OH neurons upon DT administration. The orexin promoter was obtained from a previously published construct [S3]. We exchanged the GFP cDNA fragment with a 632 bp HB-EGFP cDNA SalI-NotI fragment amplified from orfeome clone HAIB:100067676 (Geneservice). A 3.3 kb XhoI-BfrI fragment was excised and microinjected into pronuclei from B6D2 mice by standard methods. Transgenic founder mice were identified by PCR. Expression of functional DT receptors was validated by injection of DT followed by counting of immunoreactive OH neurons. Complete absence of OH neurons in the hypothalami of OH-DTR mice treated with DT confirmed transgenic expression in all of the OH cells. Following breeding and characterization we picked one of two phenotypically similar founder lines for all subsequent studies.

Food intake monitoring and pair-feeding

Adult male mice were housed individually in metabolic cages equipped with automated anti-spill food-weighing devices (TSE PhenoMaster), and allowed to acclimatise for one week before the experiments, during which eating was measured and confirmed to reach a steady state. In the food hopper, the food could only be eaten by standing on a grating below the food source; any food spillage was thus captured by the grating and not counted as food intake. We have performed experiments to determine the TSE PhenoMaster sensitivity in our experimental room (since the sensitivity can depend on room vibration), which demonstrated that the system is accurate to 0.01g with a mean standard error of less than 0.008 g (Fig. S4A-D). This directly confirms that the food intake changes we discovered (≈0.2 g) are well above the range of measurement error. Diet was standard chow (LabDiet 5021). Animals were kept on a 12h light-dark cycle (lights on at 7am). Room temperature was 22°C. In circadian analysis of behaviour, the time is given as 24h Zeitgeber (ZT) time, where ZT0 corresponds to lights on time, and ZT12 corresponds to lights off. Body weight was measured manually on a daily basis at the end of the lights-on period (between ZT10–ZT11). Diphtheria toxin (DT) (Sigma D0564) was diluted to 1 μg/ml and injected i.p.; two DT doses (at days 0 and 2) of 100 ng each were given. The animals were between 7 and 12 weeks old on the day of DT injection.

The extent to which excessive body weight gain is due to increased food intake was investigated using a previously described pair-feeding paradigm [S10]. In the pair-feeding experiments, the animals were individually housed in metabolic cages and injected with DT as described above. The pair-feeding protocol was started one week after DT injection because it takes several days for DT to have an effect on OH cell count (Fig. 2C). The DTR(-) mice had free access to food and their intake was measured automatically (PhenoMaster). The amount of food consumed by each of the DTR(-) animals during a 24-h period was then given its DTR(+) littermate during the following 24 hours. For food restriction experiments (Fig. 3), the food was removed for 24 hours at the end of the light period (ZT10–ZT11). Water access was not restricted.

Histology

The mice were perfused with PBS followed by 4% PFA, and the brains were removed and placed in 30% sucrose in PBS for cryopreservation. The brains were then frozen on dry ice with OCT compound and stored at −80°C until needed. 30 μm-thick coronal sections of the lateral hypothalamus were cut with a cryostat (Leica CM3050 S). The sections were stored in cryoprotectant at −20°C until needed.

Of these, every sixth section was taken for immunohistochemistry, which was performed with overnight incubation with a goat anti-orexin primary antibody (1:2000, Santa Cruz Biotechnology sc-8070), followed by a 2 hr incubation with anti-goat Alexa Fluor 488 (1:400; Invitrogen A11055) secondary antibody. To confirm the presence of MCH neurons we used a rabbit anti-MCH primary antibody (1:2000; Phoenix Pharmaceuticals H-070-47) and an anti-rabbit Alexa 568 secondary (1:400; Invitrogen A11011). Hoechst was used for nuclear counter-staining. Full-section images were captured semi-automatically with an Olympus virtual slide scanning micro- scope (VS120, 10x objective). Fiji open source software was used for image processing and the cells were counted manually using the PointPicker plugin.

Glucose tolerance tests

Intraperitoneal glucose tolerance tests were performed in DTR(+) and DTR(-) littermates after an overnight fasting period (~18h fast). A 25% aqueous glucose solution was administered by i.p. injection at a dose of 1g glucose per kg weight [S11]. Blood samples were taken from the tip of the tail 0, 15, 30, 60 and 120 min later (Fig. S4E), and glucose was measured with a blood glucose monitor (Accu-Chek Aviva Nano, Roche Diagnostics). The area under the curve (AUC; Fig. S4E) above baseline was calculated using the trapezoid rule; an unpaired *t* test was used to test for statistical significance.

Data analysis

Data were analyzed and plotted with R 3.2.0 (R Core Team, 2015), Prism 6 (GraphPad software), or Origin 2015 (Microcal). Statistical significance was tested as described in the figure legends, and $p < 0.05$ was considered significant. We used bootstrap methods [S12] to test the hypothesis that licking correlates with changes in GCaMP6s signals (Fig 1G). Values of fluorescence obtained during licking were randomly sampled (with replacement) and their mean was calculated. The same was done with samples obtained during non-licking, and this procedure was repeated 10000 for each of the two data sets. The distribution of bootstrap means is illustrated in Fig 1G, top, whereas Fig 1G, bottom, shows the distribution of the differences of the bootstrap mean values. In all cases these differences were above 0 (for Fig 1G, bottom, the mean difference was 4.87% and 95% confidence interval was [4.75, 4.98]), showing that GCaMP6s fluorescence increased with licking and that this increase was unlikely to occur by chance.

SUPPLEMENTAL REFERENCES

S1. Schöne, C., Cao, Z.F., Apergis-Schoute, J., Adamantidis, A., Sakurai, T., and Burdakov, D. (2012). Optogenetic probing of fast glutamatergic transmission from hypocretin/orexin to histamine neurons in situ. J. Neurosci. *32*, 12437-12443.

S2. Matsuki, T., Nomiyama, M., Takahira, H., Hirashima, N., Kunita, S., Takahashi, S., Yagami, K., Kilduff, T.S., Bettler, B., Yanagisawa, M., et al. (2009). Selective loss of GABA(B) receptors in orexin-producing neurons results in disrupted sleep/wakefulness architecture. Proc. Natl. Acad. Sci. U S A *106*, 4459-4464.

S3. Burdakov, D., Jensen, L.T., Alexopoulos, H., Williams, R.H., Fearon, I.M., O'Kelly, I., Gerasimenko, O., Fugger, L., and Verkhratsky, A. (2006). Tandem-pore K+ channels mediate inhibition of orexin neurons by glucose. Neuron *50*, 711-722.

S4. Chen, T.W., Wardill, T.J., Sun, Y., Pulver, S.R., Renninger, S.L., Baohan, A., Schreiter, E.R., Kerr, R.A., Orger, M.B., Jayaraman, V., et al. (2013). Ultrasensitive fluorescent proteins for imaging neuronal activity. Nature *499*, 295-300.

S5. Velez-Fort, M., Rousseau, C.V., Niedworok, C.J., Wickersham, I.R., Rancz, E.A., Brown, A.P., Strom, M., and Margrie, T.W. (2014). The stimulus selectivity and connectivity of layer six principal cells reveals cortical microcircuits underlying visual processing. Neuron *83*, 1431-1443.

S6. Jego, S., Glasgow, S.D., Herrera, C.G., Ekstrand, M., Reed, S.J., Boyce, R., Friedman, J., Burdakov, D., and Adamantidis, A.R. (2013). Optogenetic identification of a rapid eye movement sleep modulatory circuit in the hypothalamus. Nat. Neurosci. *16*, 1637-1643.

S7. Perkins, K.L. (2006). Cell-attached voltage-clamp and current-clamp recording and stimulation techniques in brain slices. J. Neurosci. Methods *154*, 1-18.

S8. Gunaydin, L.A., Grosenick, L., Finkelstein, J.C., Kauvar, I.V., Fenno, L.E., Adhikari, A., Lammel, S., Mirzabekov, J.J., Airan, R.D., Zalocusky, K.A., et al. (2014). Natural neural projection dynamics underlying social behavior. Cell *157*, 1535-1551.

S9. Hayar, A., Bryant, J.L., Boughter, J.D., and Heck, D.H. (2006). A low-cost solution to measure mouse licking in an electrophysiological setup with a standard analog-to-digital converter. J. Neurosci. Methods *153*, 203-207.

S10. Ellacott, K.L., Morton, G.J., Woods, S.C., Tso, P., and Schwartz, M.W. (2010). Assessment of feeding behavior in laboratory mice. Cell. Metab. *12*, 10-17.

S11. Ayala, J.E., Samuel, V.T., Morton, G.J., Obici, S., Croniger, C.M., Shulman, G.I., Wasserman, D.H., McGuinness, O.P., and Consortium, N.I.H.M.M.P.C. (2010). Standard operating procedures for describing and performing metabolic tests of glucose homeostasis in mice. Dis. Model. Mech. *3*, 525-534.

S12. Calmettes, G., Drummond, G.B., and Vowler, S.L. (2012). Making do with what we have: use your bootstraps. J. Physiol. *590*, 3403-3406.

Legend of Supplemental Video (related to Figure 1)

Real-time recording of orexin/hypocretin population activity (top left) during a continuous licking bout in a freely-moving mouse. Arrow shows the location of food spout containing liquid food (strawberry milkshake). Red dot marks the time when the mouse starts licking.
